# Supplementary material for: Little millet genome reveals evolutionary insights into tetraploid structure and genetic basis of micronutrient density
Source: Nat Commun. 2025 Nov 29;17:74. doi: 10.1038/s41467-025-66716-6 (PMC12769566; doi:10.1038/s41467-025-66716-6)
Supplement: Supplementary file 1 — Supplementary Information [file 41467_2025_66716_MOESM1_ESM.pdf]

Little millet genome reveals evolutionary insights into tetraploid structure and genetic basis of micronutrient density

Gali *et al*.

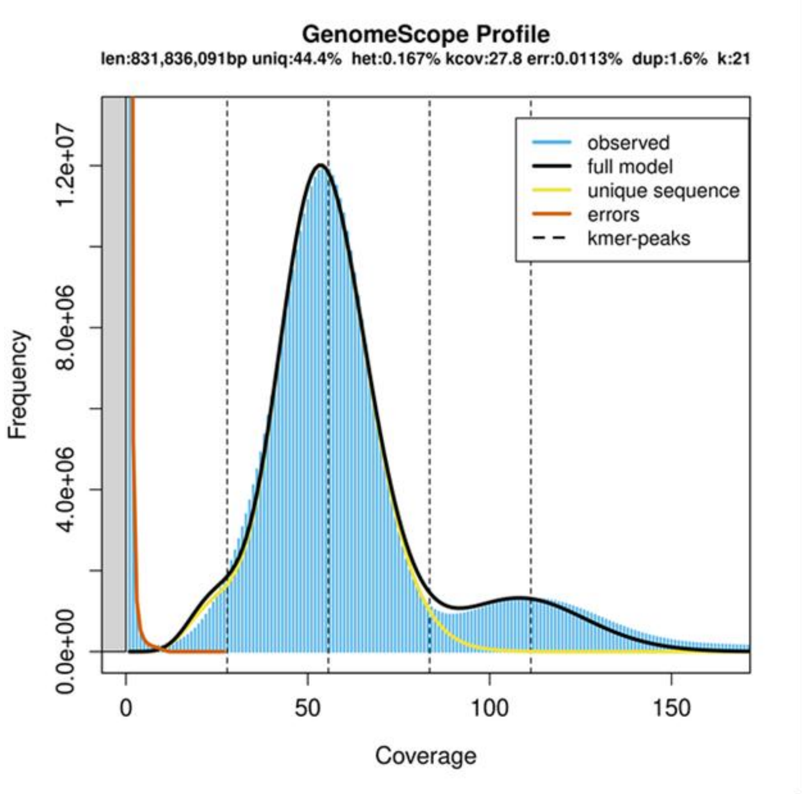

Supplementary Figure 1. K-mer-based genome size estimation of little millet. The graph depicts the k-mer frequency distribution obtained from PacBio HiFi data, analyzed with a k-mer size of 21. The x-axis represents the k-mer coverage, and the y-axis shows the frequency of k-mers with a given coverage. The main peak corresponds to the homozygous k-mer coverage, while the smaller peaks indicate repetitive regions and heterozygous loci. The fitted model (solid line) generated by GenomeScope estimates the genome size, heterozygosity, and repeat content. Key estimated parameters include a genome size of approximately 831.8 Mb and heterozygosity rate of 0.167%.

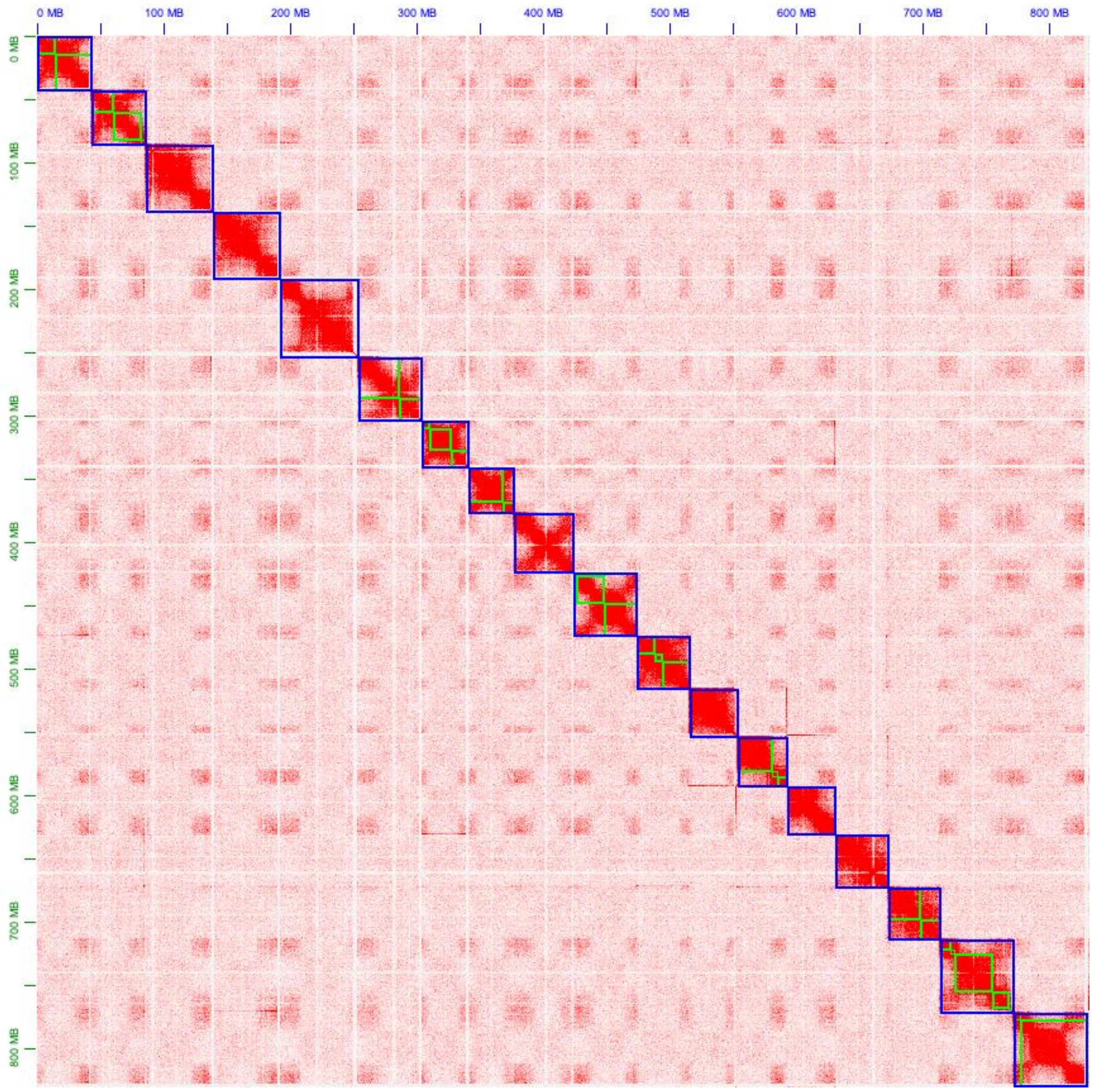

Supplementary Figure 2. Hi-C contact map of the little millet genome. The heatmap illustrates the Hi-C interaction frequencies across the chromosomes of little millet. The x-axis and y-axis represent genomic coordinates along the chromosomes, with each square indicating the frequency of physical interactions between genomic regions. Darker shades represent higher interaction frequencies, typically corresponding to regions of closer spatial proximity in the nucleus. Chromosomal boundaries and the formation of topologically associating domains (TADs) are evident, highlighting the three-dimensional organization of the genome. This map provides insights into chromosomal scaffolding and genome assembly accuracy.

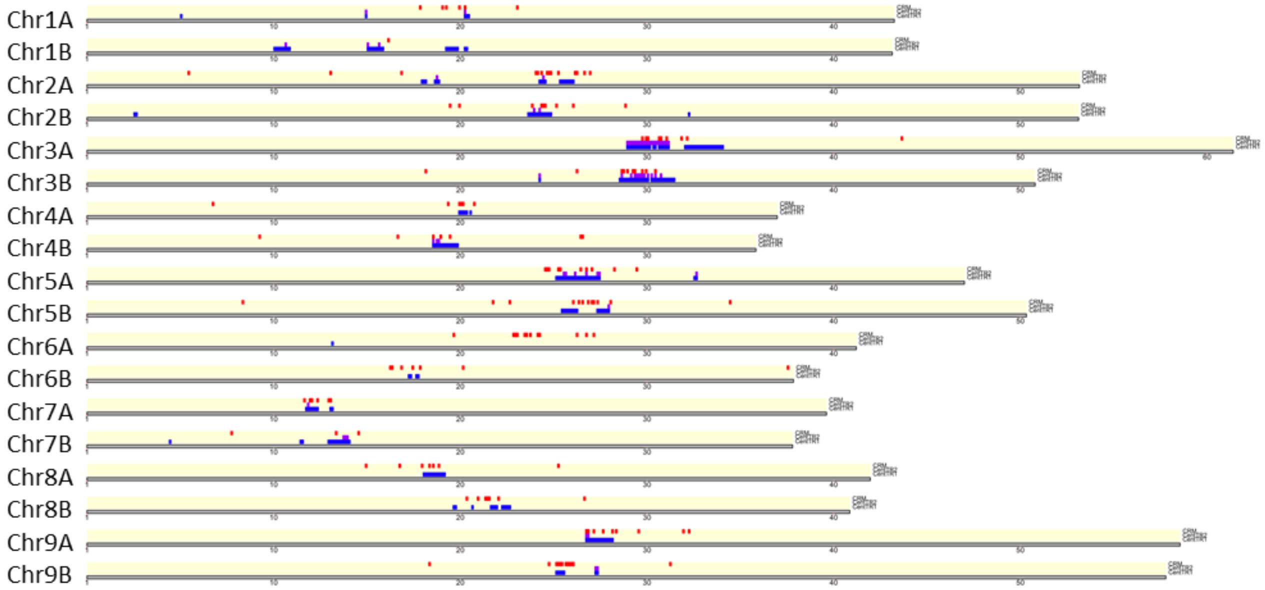

Supplementary Figure 3. Genomic distribution of centromere-associated tandem repeats (CentTR1 and CentTR2) and centromeric retrotransposons in little millet, delineating putative centromeric and pericentromeric regions.

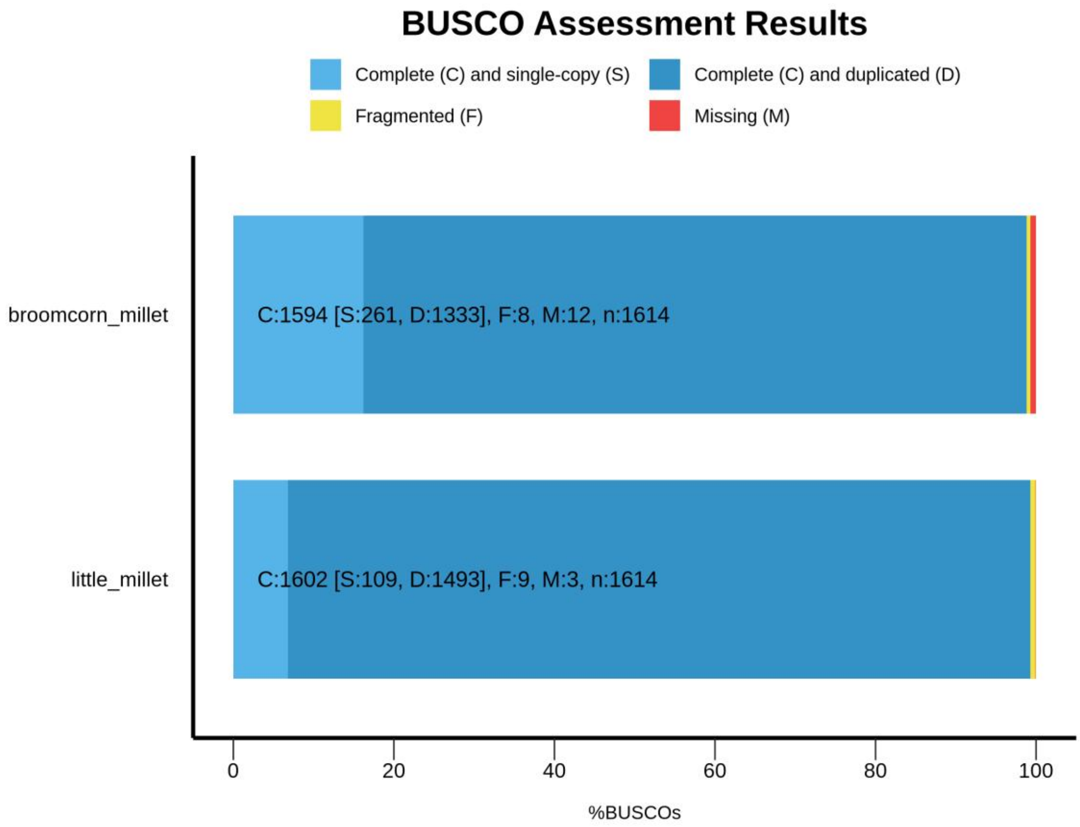

Supplementary Figure 4. BUSCO results of little millet and broomcorn millet genomes based on the embryophyta database. The bar chart summarizes the completeness of little millet and broomcorn millet genome assemblies as evaluated by Benchmarking Universal Single-Copy Orthologs (BUSCO). The assessment categorizes orthologs into four groups: complete and single-copy (sky blue), complete and duplicated (blue), fragmented (yellow), and missing (red). Results are benchmarked against the Embryophyta lineage, representing land plant genomes.

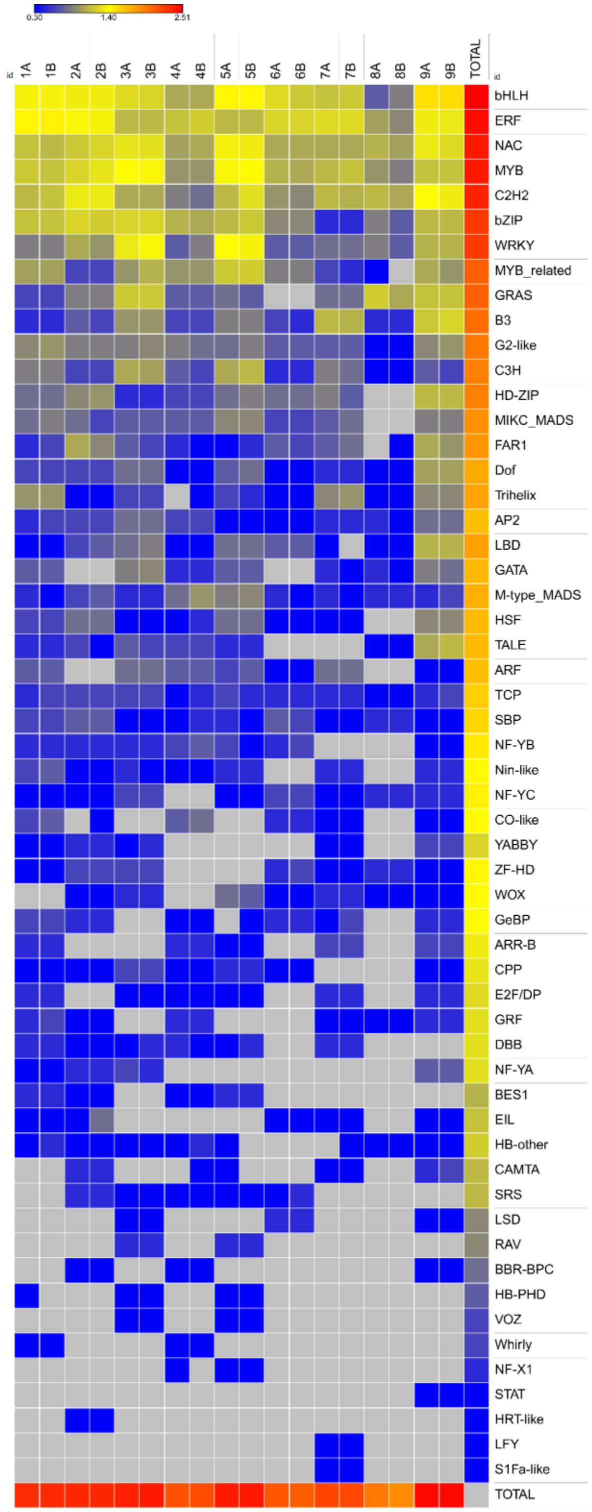

Supplementary Figure 5. Transcription Factor gene families in little millet.

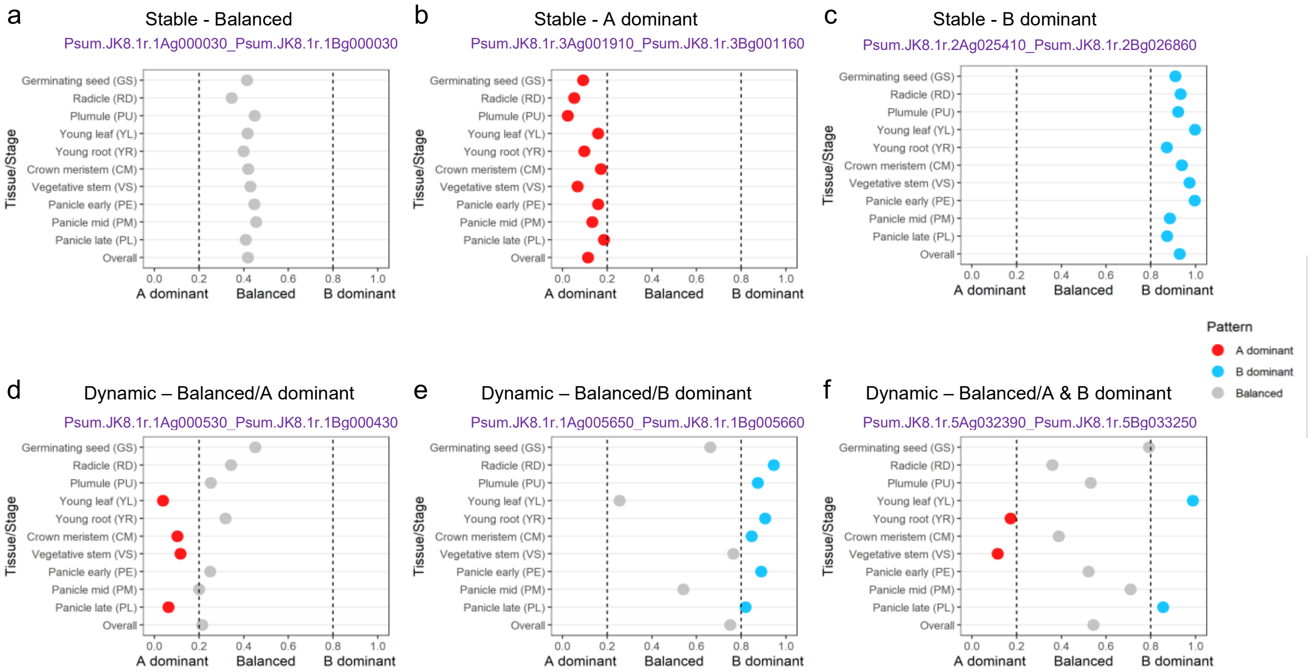

Supplementary Figure 6. Plots of representative stable and dynamic gene pairs. (a)-(c) Three representative gene pairs with stable genome dominance patterns across the 10 individual tissues/stages, including (a) stable Balanced; (b) stable A dominant; and (c) stable B dominant. (d)-(f) Three representative gene pairs with dynamic genome dominance patterns across the 10 individual tissues/stages, including (d) dynamic A dominant to Balanced; (e) dynamic B dominant to Balanced; and (f) dynamic A dominant to Balanced to B dominant. x-axis, relative expression levels of B homoeologs (REb) for each gene pair used to define dominance patterns (0-0.2, A dominant; 0.2-0.8, balanced; 0.8-1, B dominant), three patterns were separated by horizontal broken lines; y-axis, different tissues from emergence, vegetative and reproductive phases. Color keys for different patterns are displayed on the right panel. Source data are provided as a Source Data file.

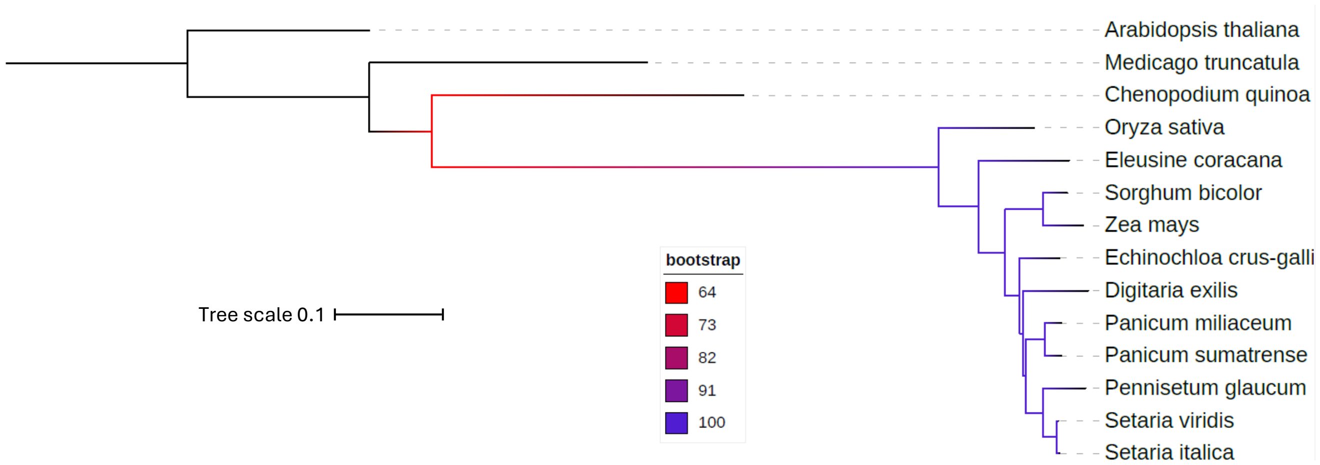

Supplementary Figure 7. Molecular Phylogeny of millet and related cereal species. A maximum likelihood tree produced from a supermatrix constructed based on 1295 orthologous sequences and a total 1,246,613 bps. Clade colors near nodes represent bootstrap proportions in percentages. Branch lengths represent estimated nucleotide substitutions per site.

A

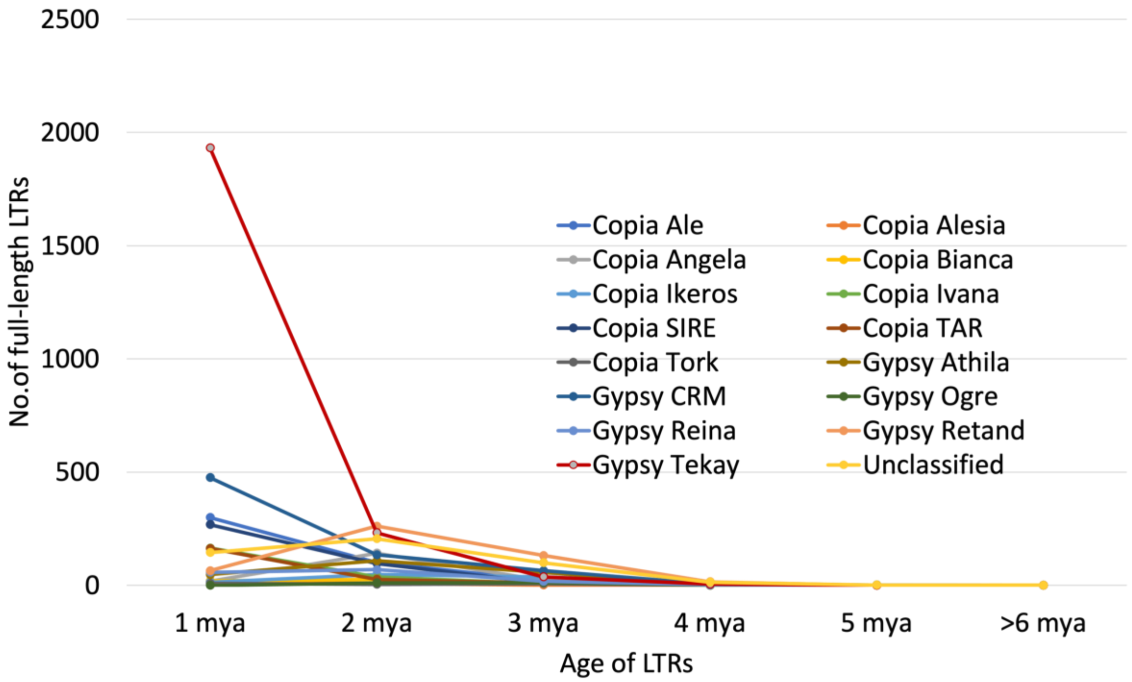

B

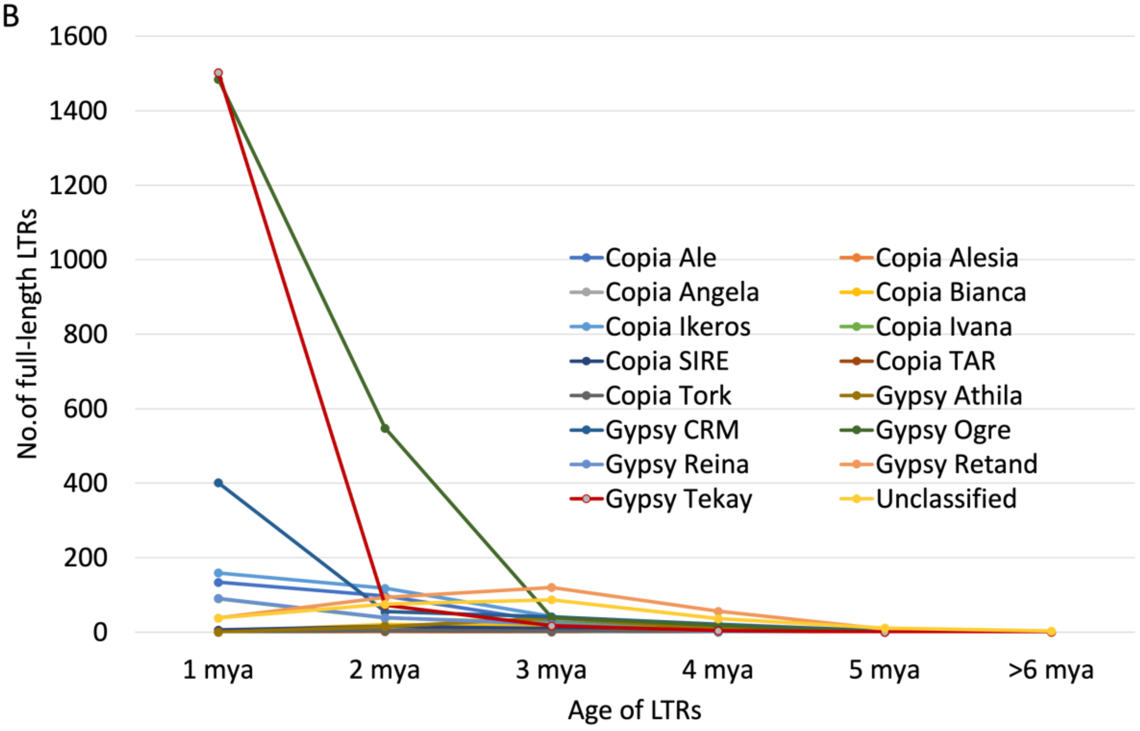

Supplementary Figure 8. Age distribution of LTRs in little millet and broomcorn millet. A. Little millet LTRs age distribution. B. Broomcorn millet LTRs age distribution. Source data are provided as a Source Data file.

A

B

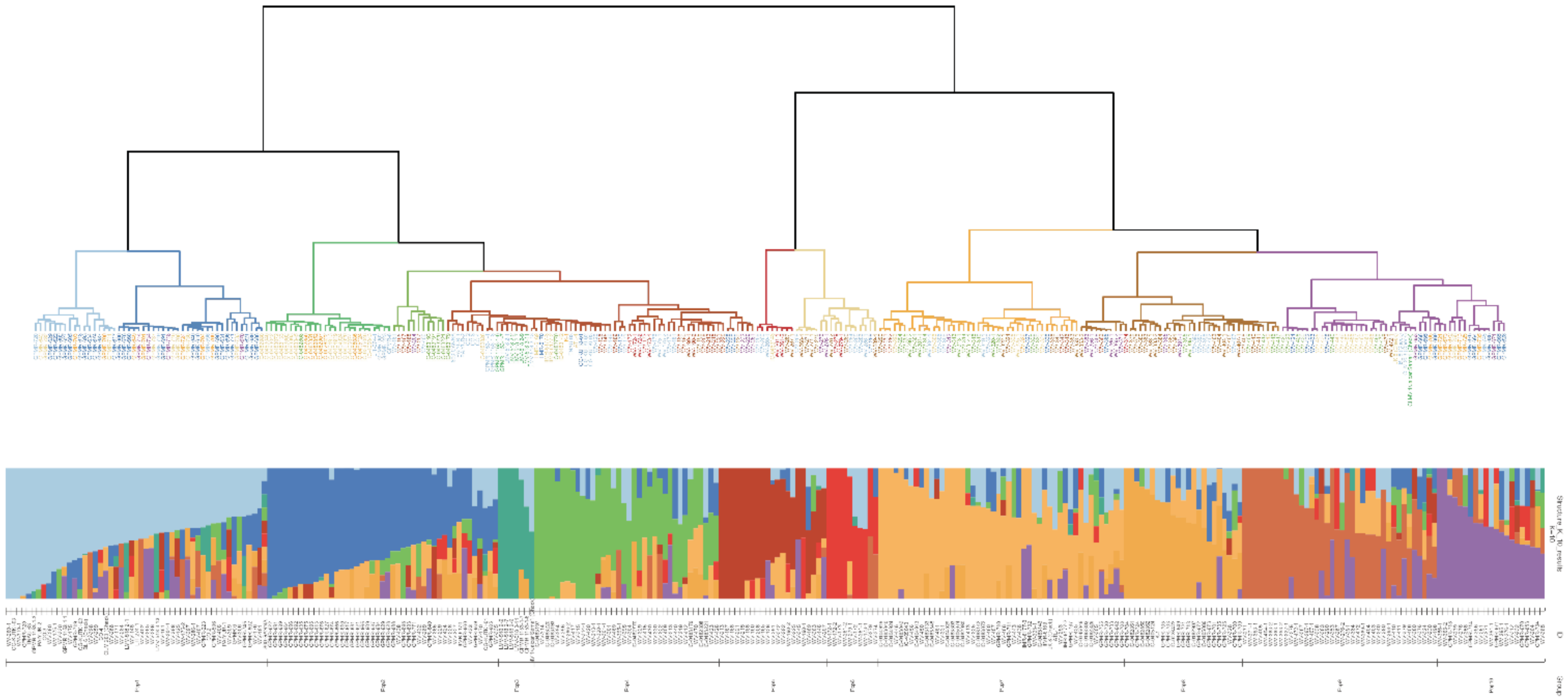

Supplementary Figure 9. Clustering of little millet diversity panel based on (A) neighbor-joining method and (B) structure analysis.

The color of little millet accessions on x-axis in Supplementary Fig. 9A match with the grouping of fastSTRUCTURE based clustering of these accessions in Supplementary Fig. 9B.

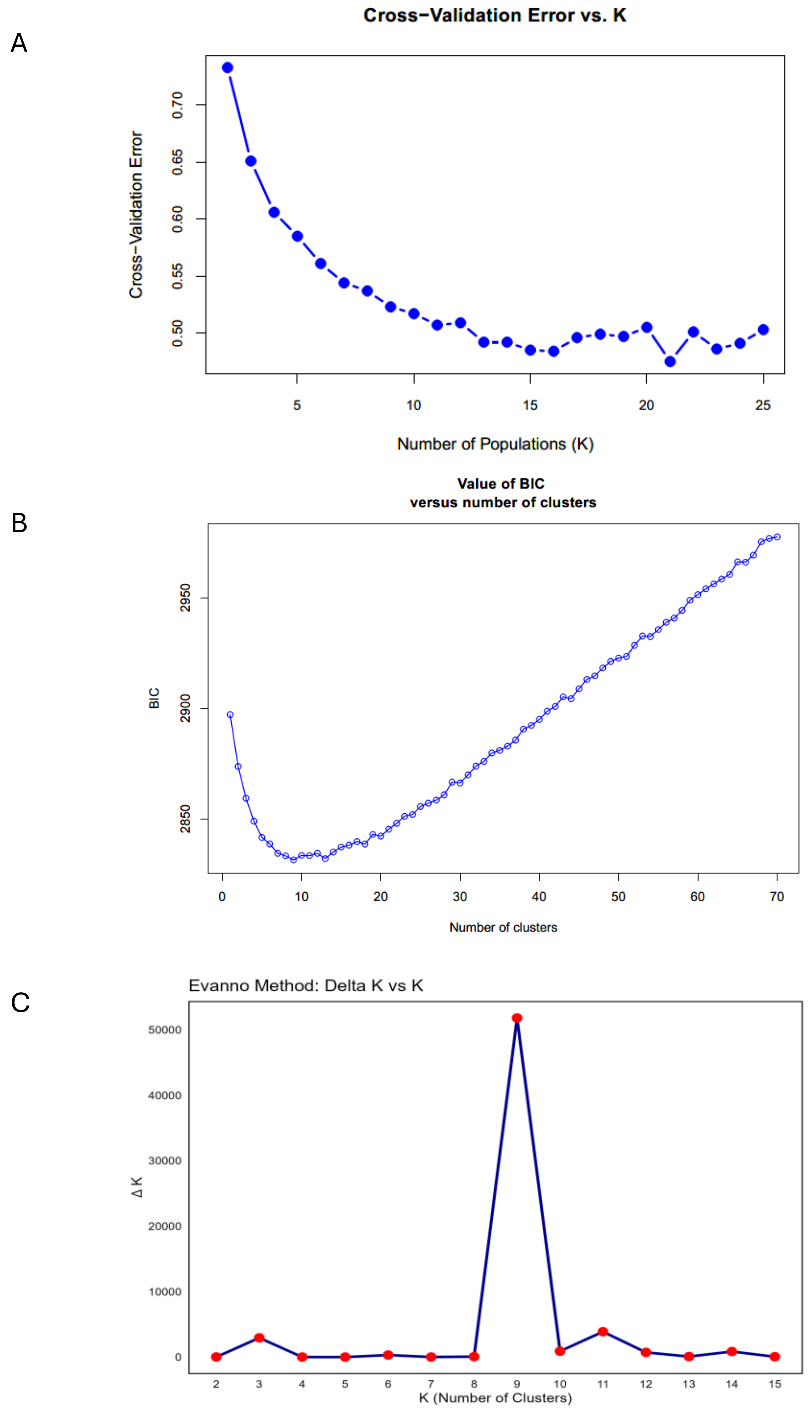

Supplementary Figure 10. Comparison of genetic grouping of little millet diversity panel determined by ADMIXTURE (A), DAPC (B), and STRUCTURE (C) models. Three hundred accessions of little millet were resequenced and 249,511 genome-wide SNPs were used for the analyses.

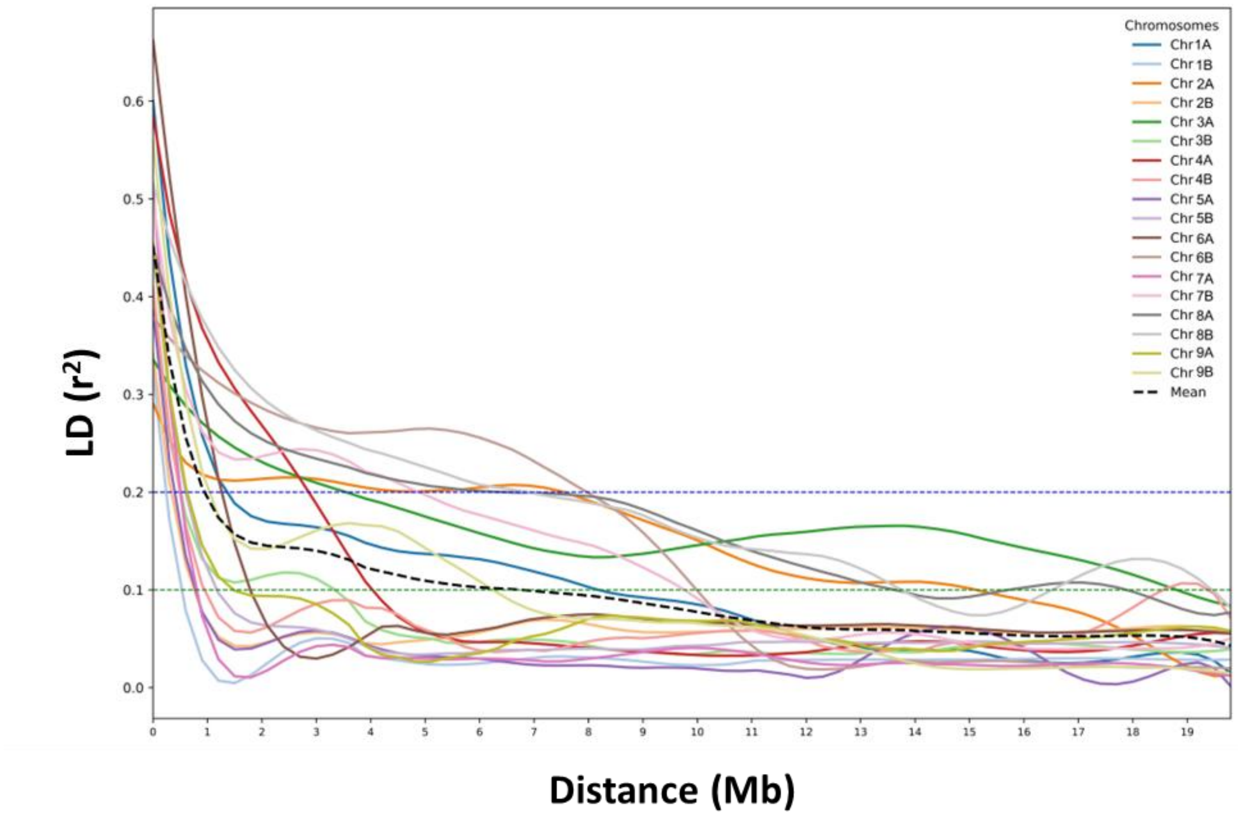

Supplementary Figure 11. Linkage disequilibrium (LD) graph of little millet. Three hundred accessions of little millet were resequenced and . 249,511 genome-wide SNPs were used to calculate LD as a function of correlation between marker-pairs calculated as Pearson correlation coefficient.

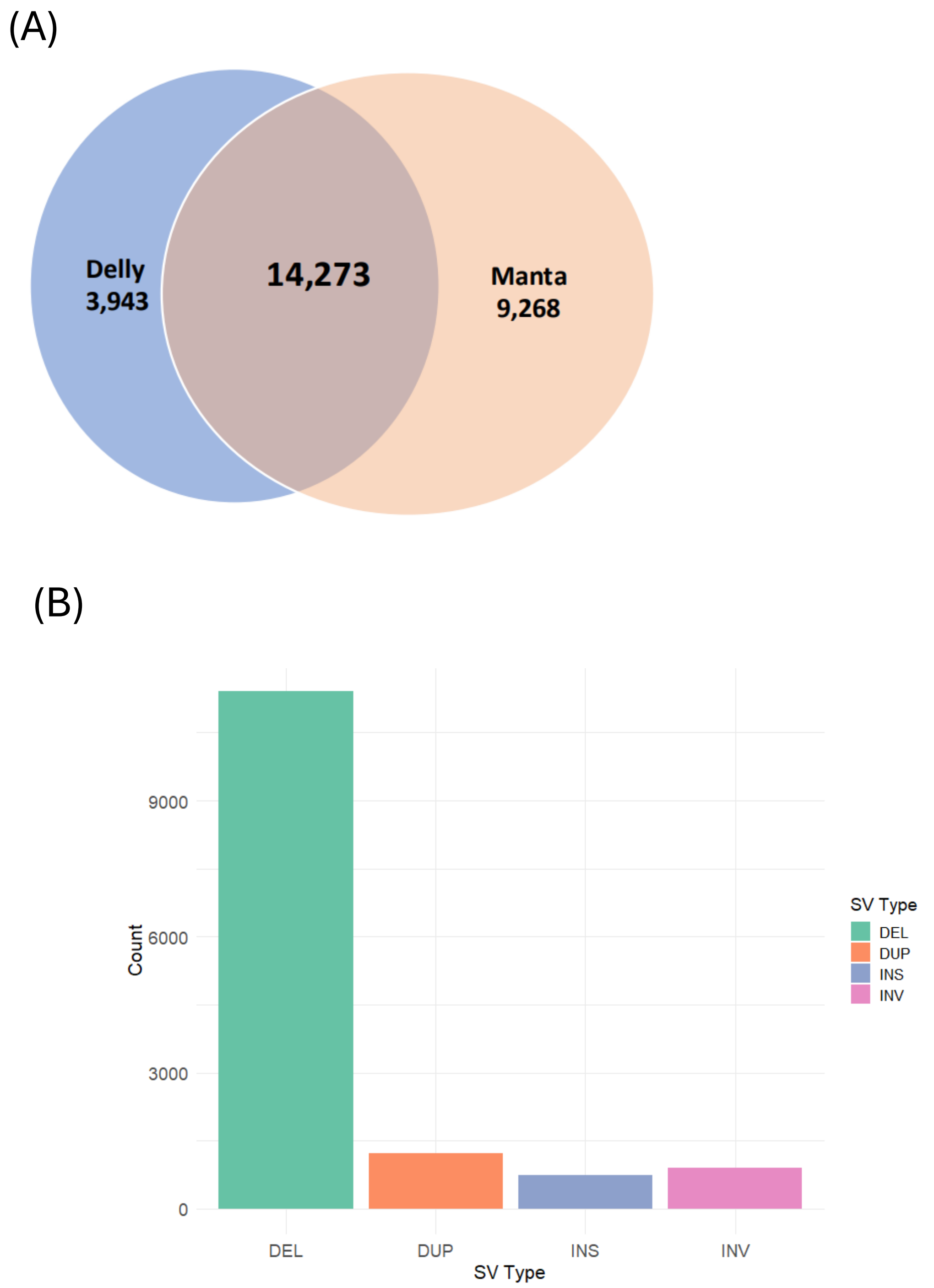

Supplementary Figure 12. Structural variants (SVs) identified from 300 little millet genomes. (A) Overlap of structural variants detected by Delly and Manta across 300 little millet genomes. Venn diagram showing shared (consensus) and tool-specific SVs. (B) SV type and size class distribution of the consensus SVs (n= 14,273). Source data are provided as a Source Data file.

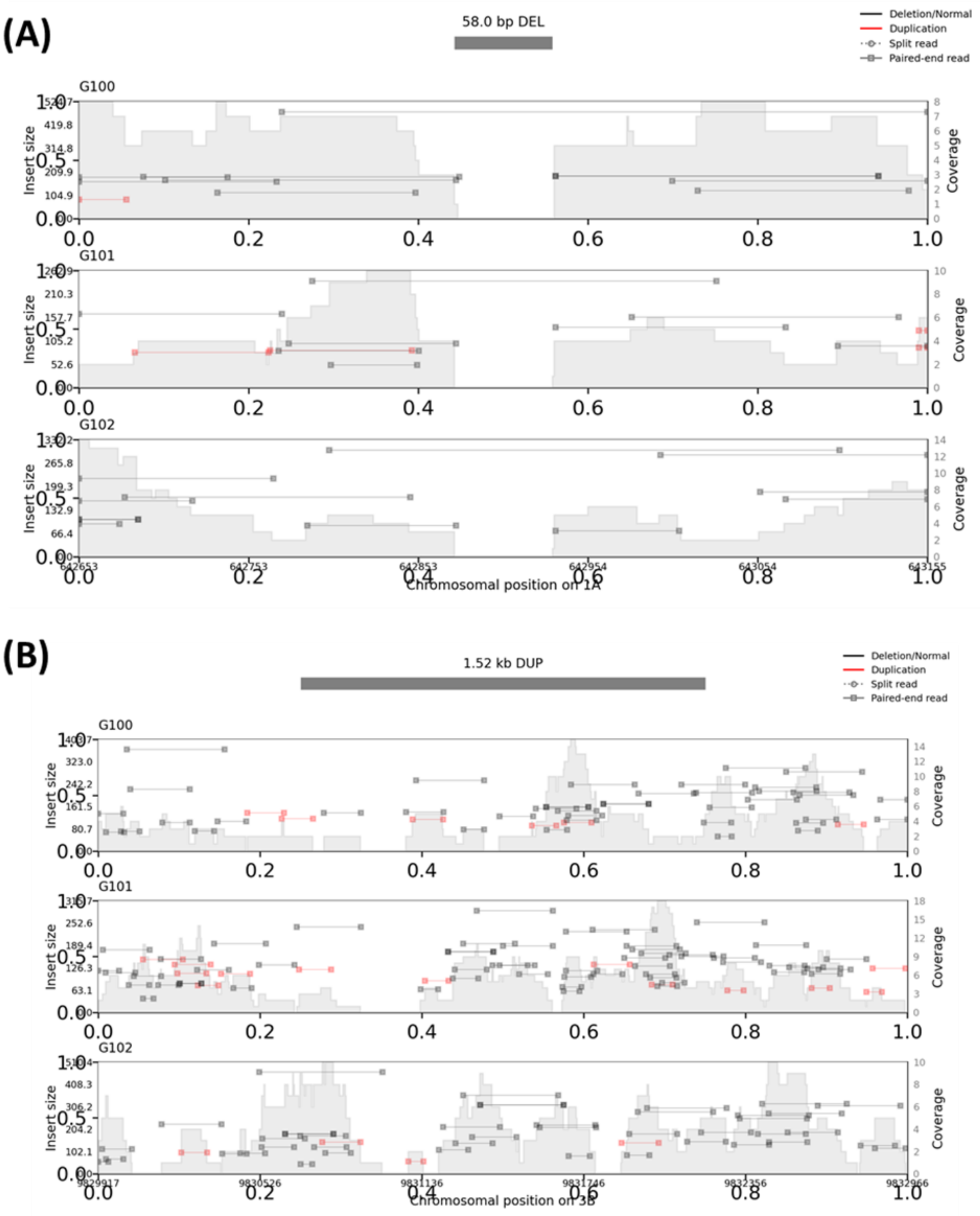

Supplementary Figure 13. Read depth-based validation of SVs using Samplot. A 58bp deletion (A) and a 1.5 kb duplication (B) identified by both Delly and Manta, showing strong read-depth support on the three lines tested.

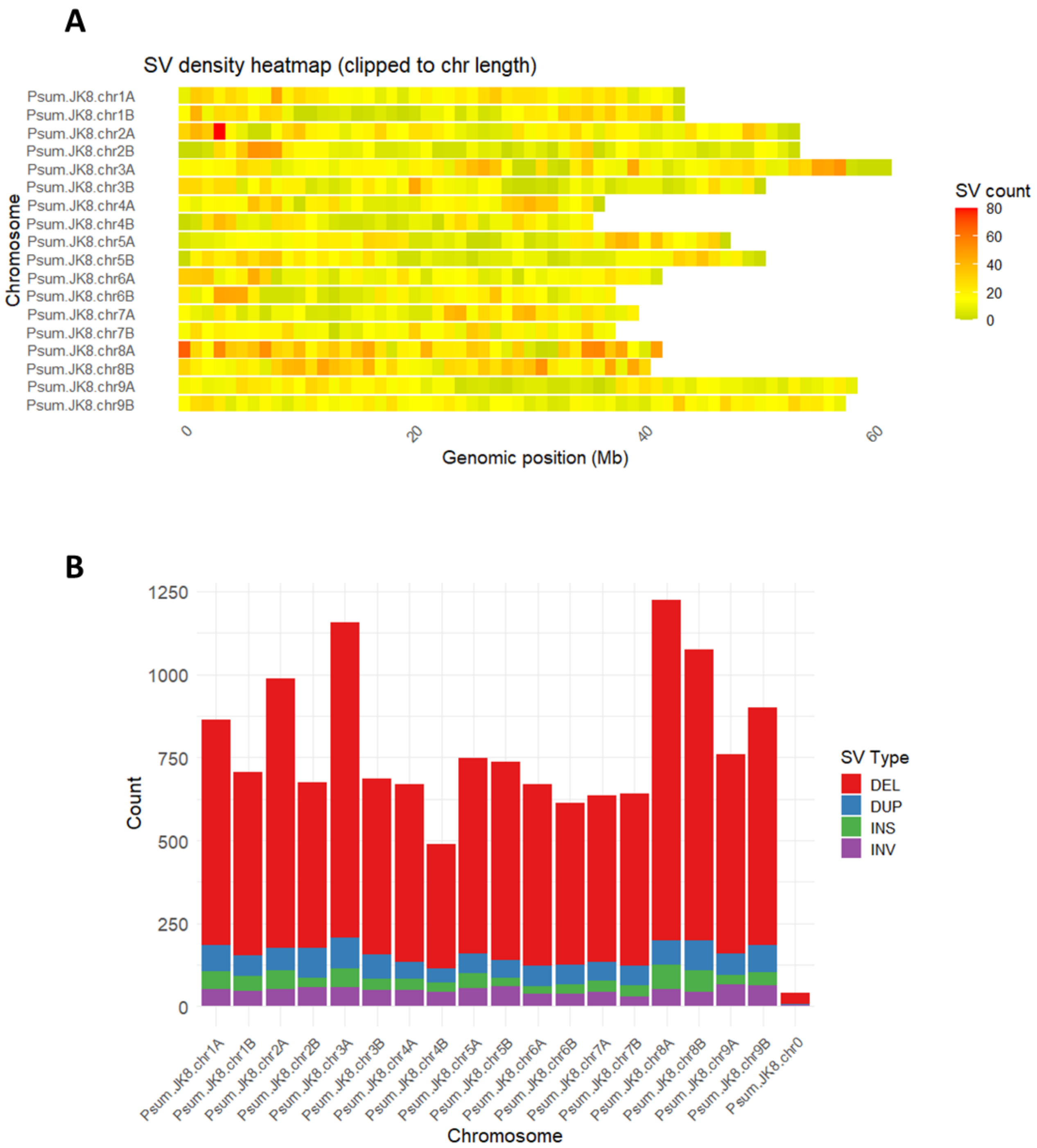

Supplementary Figure 14. Chromosome-wise distribution of structural variants (SVs) in little millet. A. Chromosomal heatmap of SV density across the 18 chromosomes. B. Distribution of SV types (DEL, DUP, INS, INV) per chromosome. Source data are provided as a Source Data file.

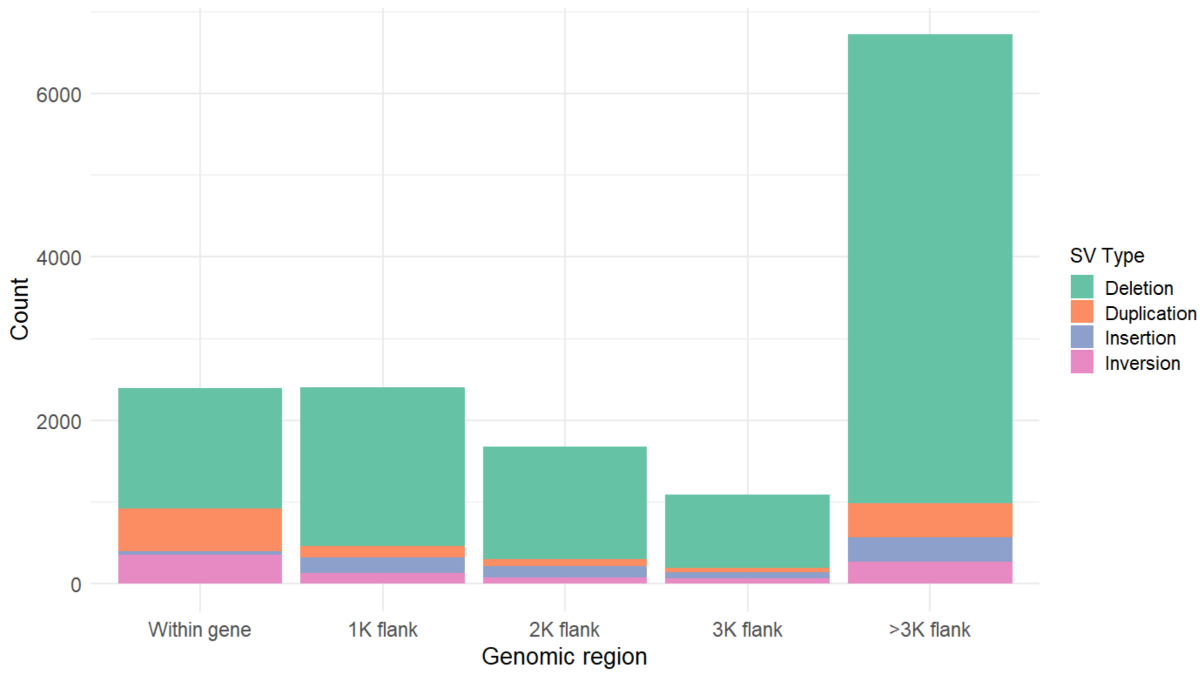

Supplementary Figure 15. Distribution of Structural variants (SVs) across genic and flanking regions (within gene body and within 1-3 kb proximity). Source data are provided as a Source Data file.

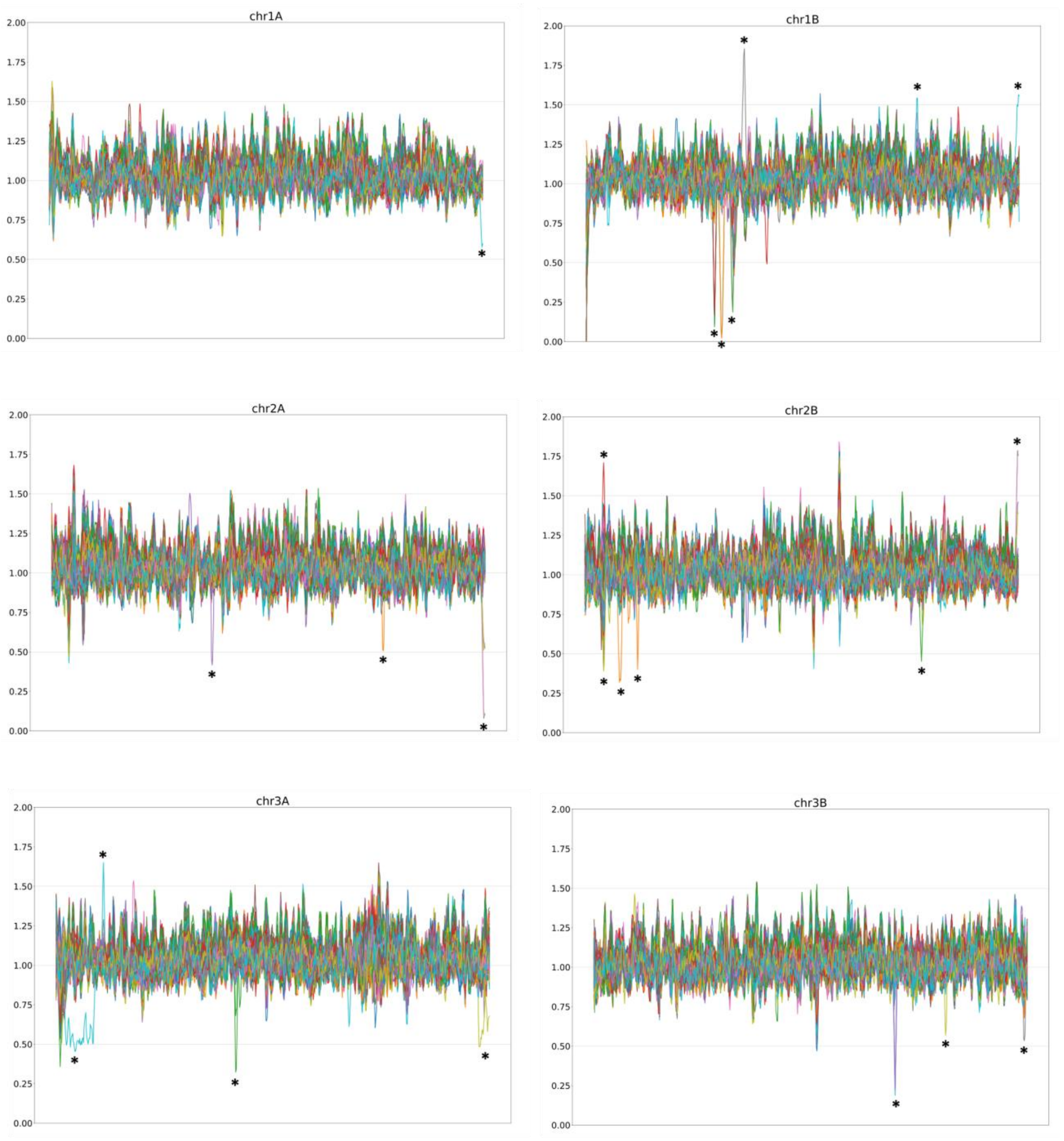

Supplementary Figure 16. LSV-viz line plots for little millet diversity panel for chromosomes 1A, 1B, 2A, 2B, 3A and 3B. Little millet cv. JK8 was used as the reference accession to calculate the gene coverage ratios for LSV-viz plotting for all other accessions. All large structural variants of interest in the diversity panel have been identified with an "*" on the plots.

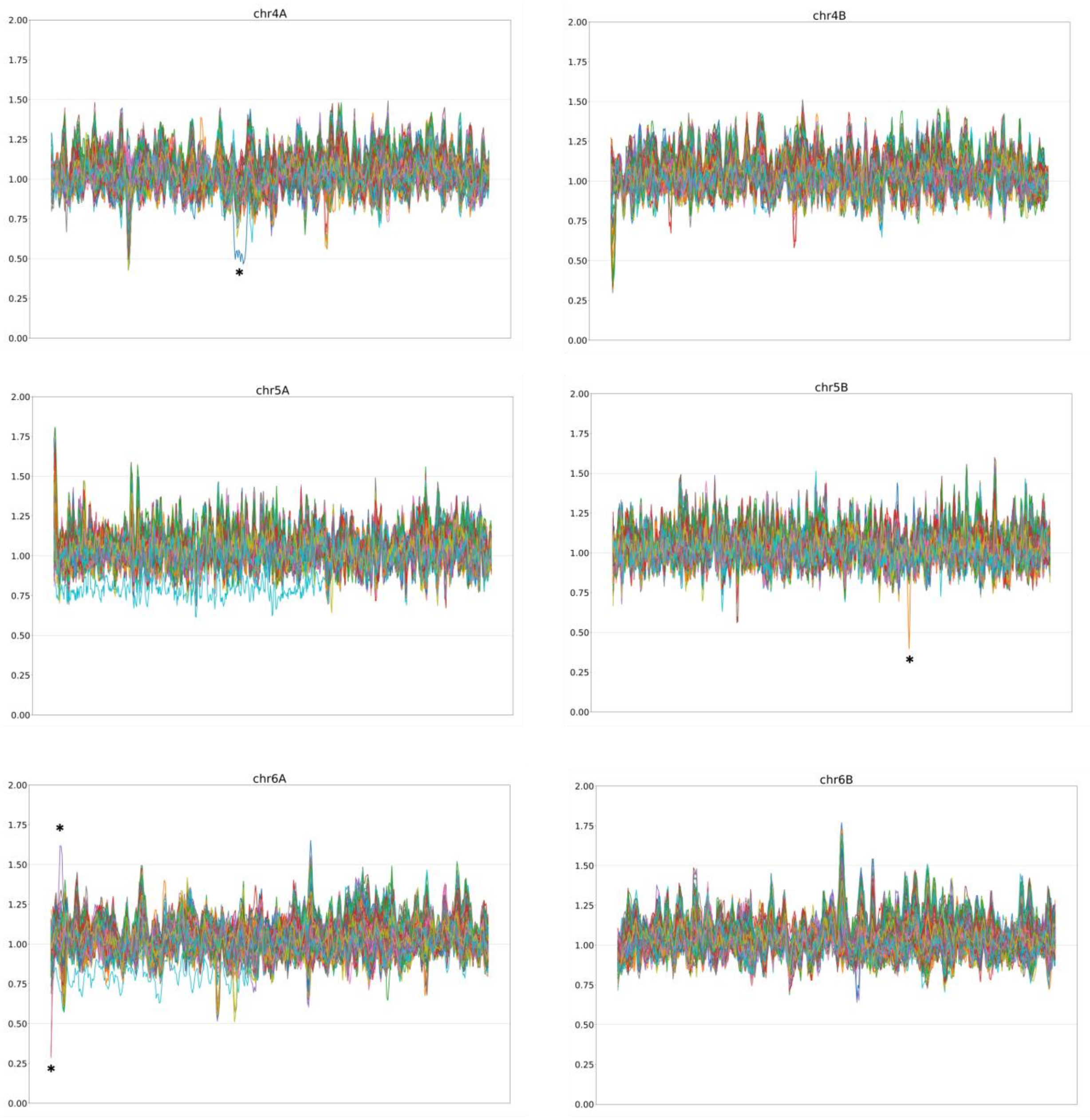

Supplementary Figure 17. LSV-viz line plots for little millet diversity panel for chromosomes 4A, 4B, 5A, 5B, 6A and 6B. Little millet cv. JK8 was used as the reference accession to calculate the gene coverage ratios for LSV-viz plotting for all other accessions. All large structural variants of interest in the diversity panel have been identified with an "*" on the plots.

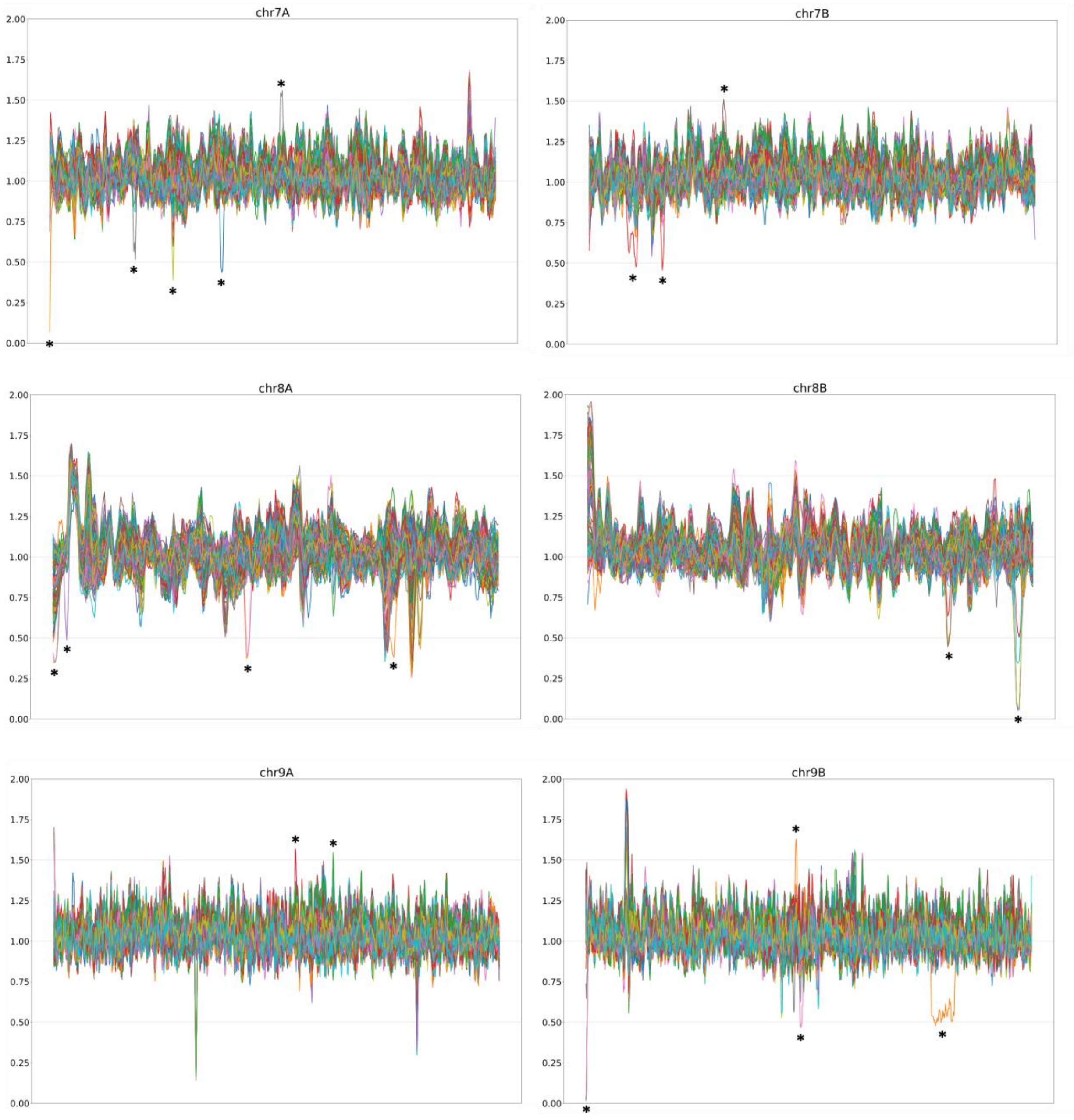

Supplementary Figure 18. LSV-viz line plots for little millet diversity panel for chromosomes 7A, 7B, 8A, 8B, 9A and 9B. Little millet cv. JK8 was used as the reference accession to calculate the gene coverage ratios for LSV-viz plotting for all other accessions. All large structural variants of interest in the diversity panel have been identified with an "*" on the plots.

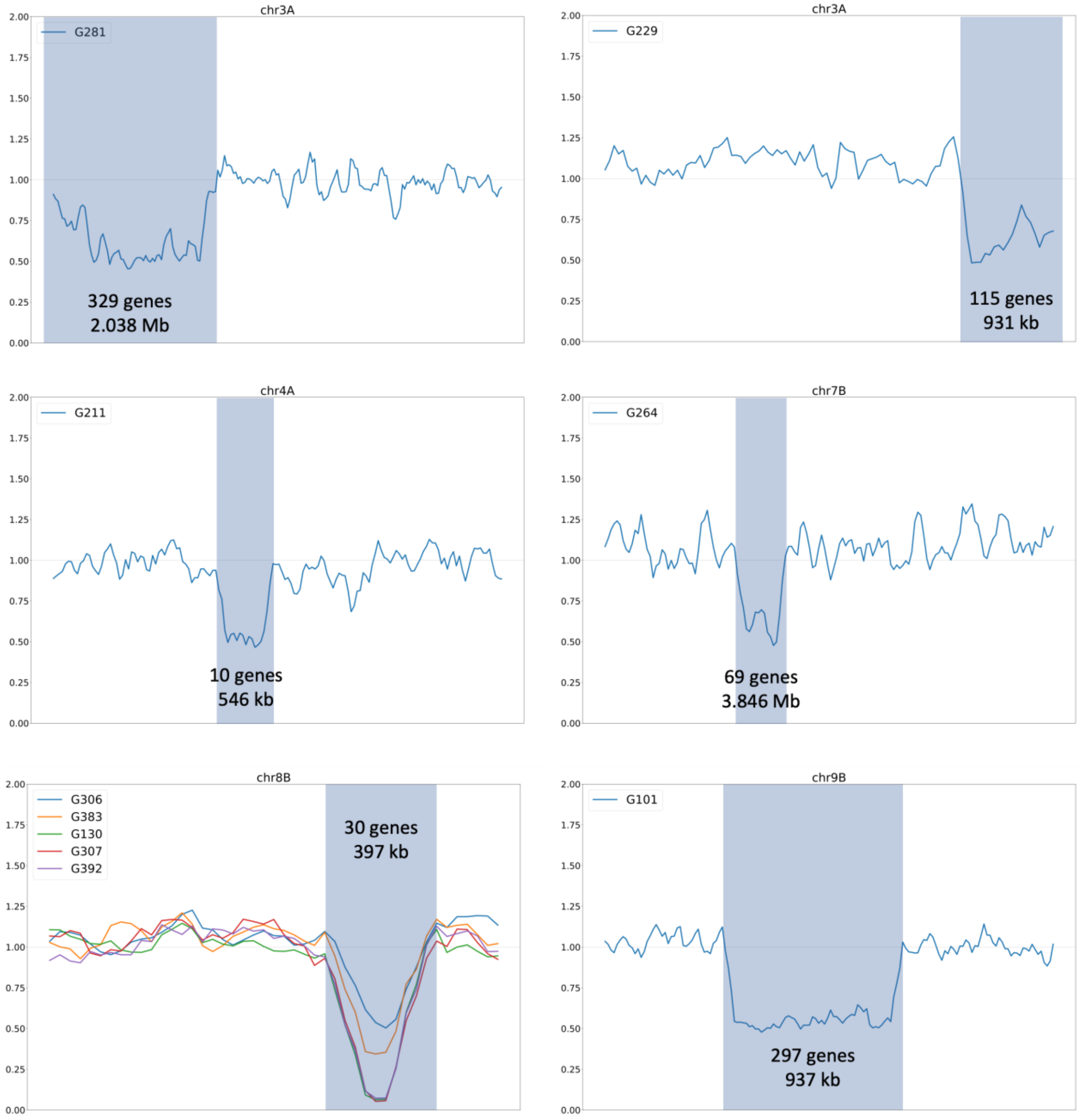

Supplementary Figure 19. Representative large structural variants. These plots show 6 representative structural variants that were identified with LSV-viz. The plots have been zoomed in to highlight the change in gene coverage ratio. Gene size and size in base pairs have been added to the plots, as well as the diversity panel line.

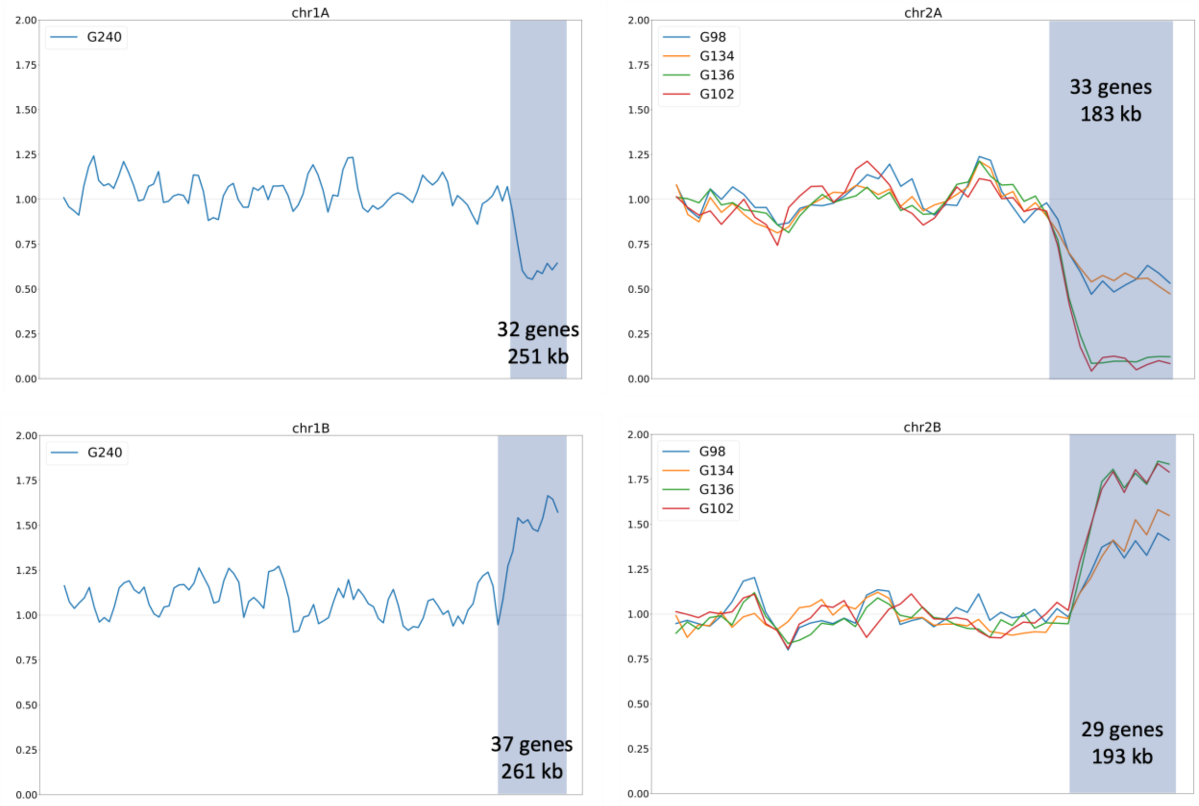

Supplementary Figure 20. Reciprocal large structural variants. Among the 300 lines in the diversity panel, there were 5 lines that were identified to have reciprocal large structural variants on homologous chromosomes. Line G240 has an approximate 0.5 drop in gene coverage ratio spanning 32 genes (~251kb) on chromosome 1A and a corresponding 0.5 increase in gene coverage ratio on 1B spanning 37 genes (~261kb) on chromosome 1B. Lines 98 and 134 have a 0.5 decrease in gene coverage ratio (33 genes, ~183kb) on chromosome 2A and a 0.5 increase in gene coverage ratio (29 genes, ~193kb) on chromosome 2B. Lines 102 and 136 show a loss of gene coverage ratio (suggesting a deletion) of 33 genes (~183kb) on chromosome 2A, and a corresponding doubling of the gene coverage ratio for 29 genes (~193kb) on chromosome 2B.

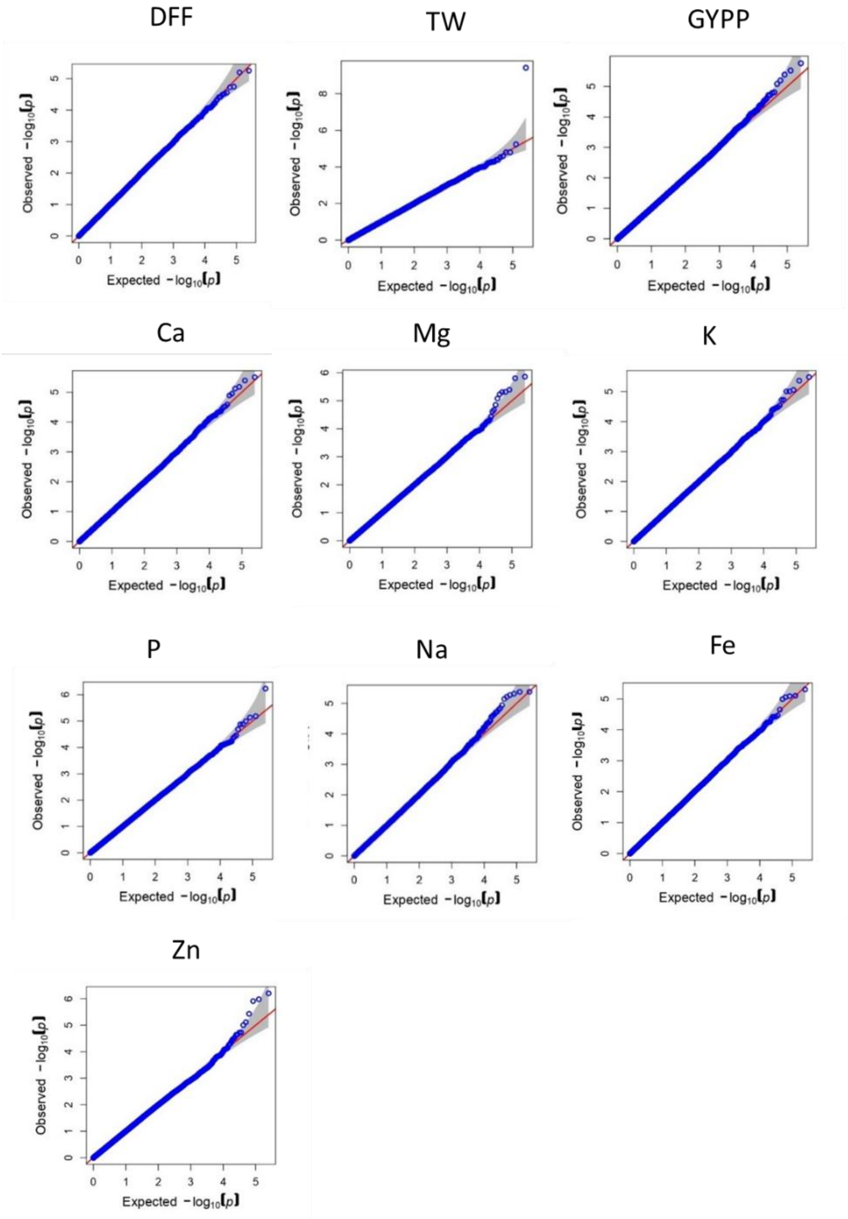

Supplementary Figure 21. Q-Q plots related to SNP-based genome-wide association mapping of agronomic traits and micronutrient concentrations of little millet. The plots compare the observed –log10(p) values on y-axis to expected –log10(p) values on x-axis. The phenotypic tests were conducted at two locations (GKVK, Bangalore and ICAR-IIMR, Hyderabad, India) and BLUPs calculated based on pooled values were used for association mapping using BLINK. The Q-Q plots represent the traits days to flowering (DFF), thousand seed weight (TW), grain yield per plant (GYPP), grain concentrations of Ca, Mg, K, P, Na, Fe and Zn.

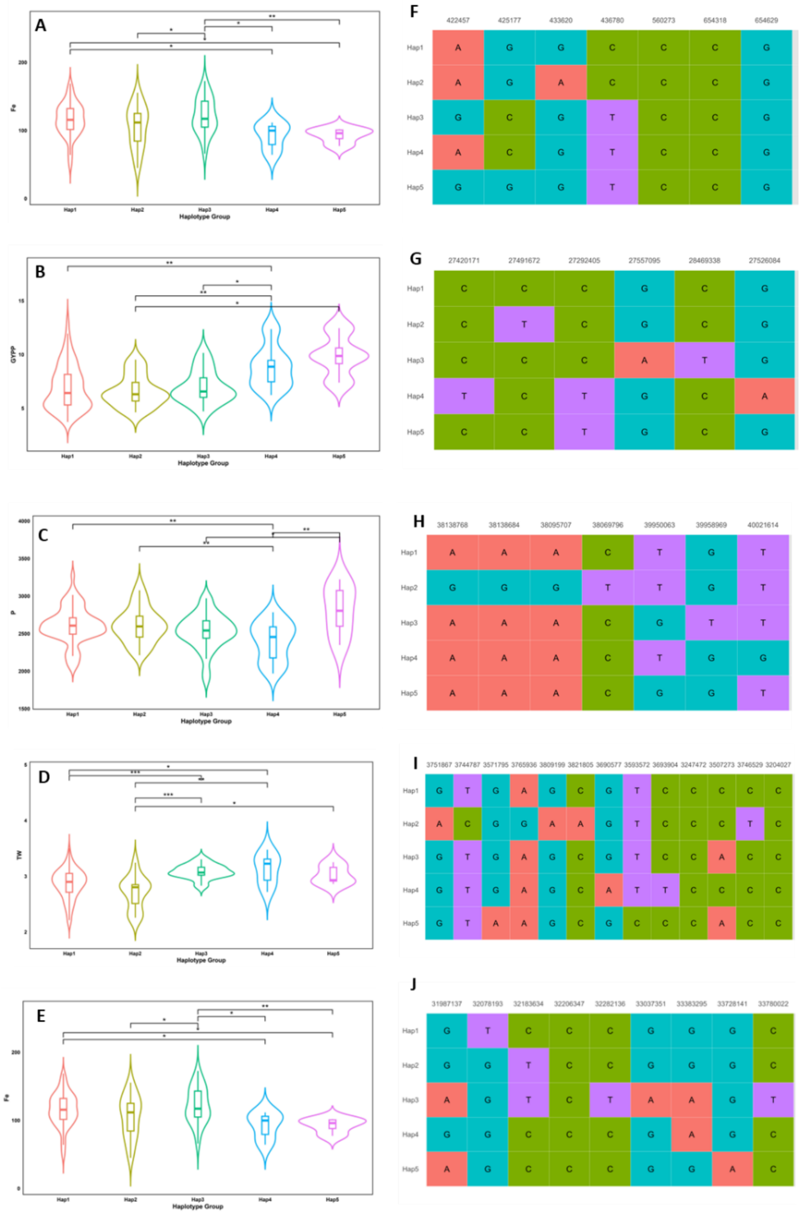

Supplementary Figure 22. Haplotype analysis of significant trait-associated SNP loci. Violin plots showing trait distribution across haplotype groups defined by SNP variation at five loci: **(A)** Chr2A_32889668 (seed iron concentration), **(B)** Chr4B_27491672 (grain yield per plant), **(C)** Chr1B_39041529 (seed P concentration), **(D)** Chr2A_3693904 (thousand seed weight), and **(E)** Chr1A_560273 (seed Fe concentration). Boxplots within violins represent medians and interquartile ranges. Asterisks denote significant differences among haplotypes ($P < 0.05$ = *, $P < 0.01$ = **, $P < 0.001$ = ***). Figures e to h represent the haplotype structure matrices which illustrate allelic patterns across a ±1 Mb region around Chr2A_32889668 **(F)**, Chr4B_27491672 **(G),** Chr1B_39041529 **(H),** Chr2A_3693904 **(I)**, and Chr1A_560273 **(J)** with the top X-axis representing chromosomal positions (SNP coordinates) and the Y-axis indicating haplotype groups. Source data are provided as a Source Data file.

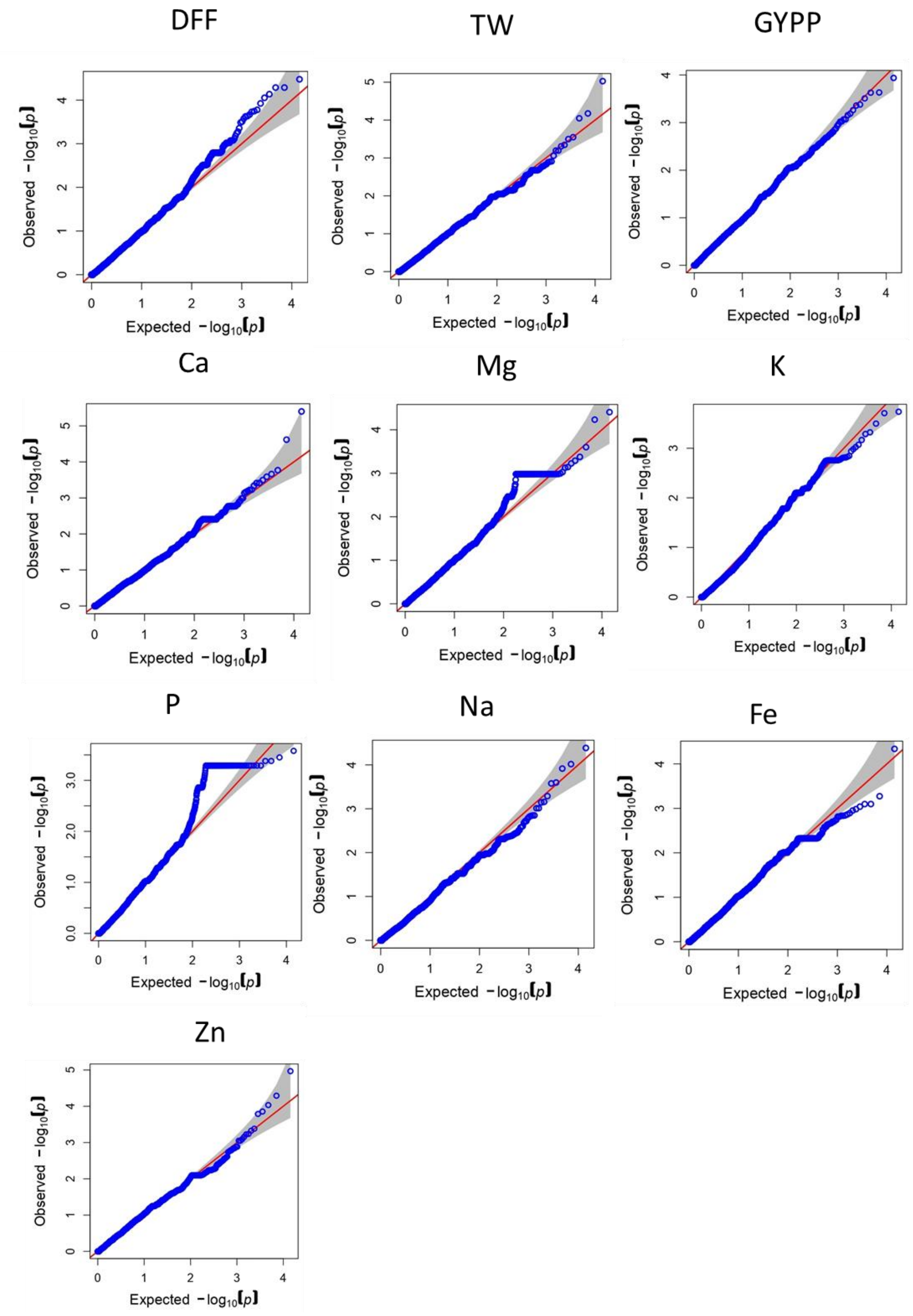

Supplementary Figure 23. Q-Q plots related to Structural variant (SVs)-based genome-wide association mapping of agronomic traits and micronutrient concentrations of little millet. The plots compare the observed –log10(p) values on y-axis to expected –log10(p) values on x-axis. The phenotypic tests were conducted at two locations (GKVK, Bangalore and ICAR-IIMR, Hyderabad, India) and BLUPs calculated based on pooled values were used for association mapping using BLINK. The Q-Q plots represent the traits days to flowering (DFF), thousand seed weight (TW), grain yield per plant (GYPP), grain concentrations of Ca, Mg, K, P, Na, Fe and Zn.

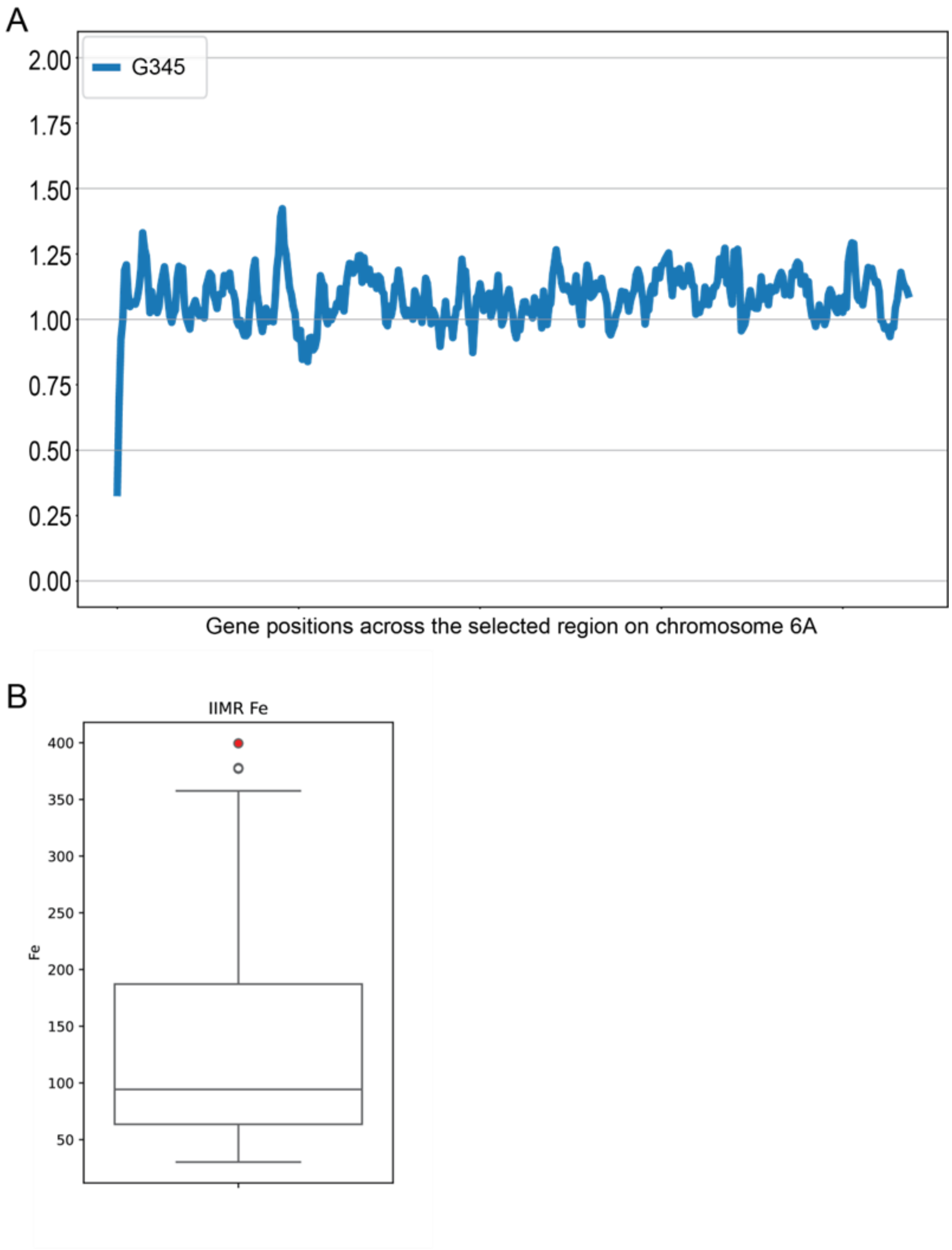

Supplementary Figure 24. LSV (deletion) identified in accession G345 (accession # WV-356-1). A. Little millet accession G345 carrying a deletion in the telomeric region. This deletion involves 4 genes. B. Iron content in G345 is almost 3x that of the check variety (JK-8). Source data are provided as a Source Data file.

Supplementary Table 1. Sequencing data generated for genome assembly of little millet.

Sequencing platform	No. of reads (Million)	Total data (Gb)	Coverage (x)
**Contig assembly**			
PacBio long reads	9.04	70.1	82.5
Nanopore long reads	42	144	169
**Hi-C based Scaffolding**			
Illumina short reads	910	273	321
**GWAS panel sequencing**			
Illumina shot reads	32 M x 330 lines	5 Gb x 330 lines	5.88 (per line)

Supplementary Table 2. Summary of little millet draft genome assembly statistics.

Description	HiFi	HiFi + ONT	HiFi + ONT + HiC (Version 1)	HiFi + ONT + HiC + gapFilled (Version 2)
No. of sequences	2019	351	18*	18*
No. of bases	883,631,838	850,600,460	850,492,432	850,463,639
Max length (bp)	53,071,109	101,412,992	61,397,489	61,397,489
N50 (bp)	26,841,802	29,408,779	47,000,000	47,000,000
L50 (Number)	12	9	8	8
N90 (bp)	6,049,143	13,018,246	36,974,821	36,974,821
L90 (Number)	32	25	17	17

*The final Pseudomolecule assembly also includes 314 unanchored contigs

Supplementary Table 3. Locations of centromeres in the little millet chromosomes.

Chromosome	Centromere start	Centromere end	Centromere size
**Chr1A**	20,100,000	20,400,000	300,000
**Chr1B**	15,100,000	18,200,000	3,100,000
**Chr2A**	24,600,000	26,800,000	2,200,000
**Chr2B**	24,200,000	26,200,000	2,000,000
**Chr3A**	29,100,000	32,200,000	3,100,000
**Chr3B**	28,600,000	31,200,000	2,600,000
**Chr4A**	19,400,000	20,200,000	800,000
**Chr4B**	16,700,000	19,200,000	2,500,000
**Chr5A**	26,100,000	29,500,000	3,400,000
**Chr5B**	25,600,000	27,200,000	1,600,000
**Chr6A**	22,500,000	23,200,000	700,000
**Chr6B**	16,200,000	17,400,000	1,200,000
**Chr7A**	11,600,000	12,900,000	1,300,000
**Chr7B**	13,300,000	14,100,000	800,000
**Chr8A**	16,100,000	18,200,000	2,100,000
**Chr8B**	20,000,000	22,100,000	2,100,000
**Chr9A**	26,700,000	28,200,000	1,500,000
**Chr9B**	25,000,000	25,500,000	500,000

Supplementary Table 4. Telomeres in the little millet chromosomes.

Chromosome	Chromosome length	Status	Left number	Left direction	Right number	Right direction
Psum.JK8.chr1A	43,243,216	left	640	+	0	
Psum.JK8.chr1B	43,137,477	both	344	+	612	-
Psum.JK8.chr2A	53,162,992	both	731	+	114	+
Psum.JK8.chr2B	53,115,015	both	1173	+	602	-
Psum.JK8.chr3A	61,397,489	right	0		823	-
Psum.JK8.chr3B	50,789,506	no	0		0	
Psum.JK8.chr4A	36,974,821	left	1647	+	0	
Psum.JK8.chr4B	35,829,799	both	947	+	918	-
Psum.JK8.chr5A	47,000,000	left	309	+	0	
Psum.JK8.chr5B	50,323,288	right	0		1466	-
Psum.JK8.chr6A	41,209,349	both	597	+	981	-
Psum.JK8.chr6B	37,846,408	right	0		450	-
Psum.JK8.chr7A	39,611,570	both	667	+	517	-
Psum.JK8.chr7B	37,794,386	right	0		202	-
Psum.JK8.chr8A	41,957,870	both	703	+	736	-
Psum.JK8.chr8B	40,861,194	right	0		710	-
Psum.JK8.chr9A	58,551,545	no	0		0	
Psum.JK8.chr9B	57,786,588	both	141	+	188	-

Telomere repeat monomers:	AAACCCT
Both telomere found:	8
Only one telomere found:	8
No telomere found:	3

Supplementary Table 5. Summary of repeat elements in the little millet genome.

		Little millet (v1)			Little millet (v2, gap-filled)			Broomcorn millet		
	Sequences:	18			18			18		
	Length:	850,492,432			850,463,639			834,678,208		
Repeat elements	Class	Count	bpMasked	%masked	Count	bpMasked	%masked	Count	bpMasked	%masked
**LTR**										
	Gypsy	206,632	281,937,638	33.15%	203,697	281,598,871	33.11%	243,231	250,050,464	29.96%
	Copia	53,271	53,567,160	6.30%	52,279	53,465,116	6.29%	41,679	38,507,726	4.61%
	Others	254	178,680	0.02%	254	178,659	0.02%	148	91,658	0.01%
**non-LTR**										
	LINE-L1	15,951	7,550,100	0.89%	15,483	7,540,197	0.89%	15,651	7,084,827	0.85%
	LINE-others	1,197	503,475	0.06%	1,196	507,733	0.06%	2,541	2,128,266	0.25%
								51,882	8,815,191	1.06%
**DNA TE**										
	DTA	16,314	4,129,600	0.49%	16,640	4,370,562	0.51%	17,552	4,159,340	0.50%
	DTC	78,772	21,539,879	2.53%	81,040	23,096,976	2.72%	87,826	26,503,665	3.18%
	DTH	40,985	8,818,911	1.04%	40,715	8,756,622	1.03%	32,381	6,894,748	0.83%
	DTM	68,438	14,820,651	1.74%	63,092	15,261,940	1.79%	50,182	11,023,908	1.32%
	DTT	63,084	10,093,846	1.19%	62,312	9,921,565	1.17%	53,851	8,972,727	1.07%
	MITE-DTA	10,045	1,381,135	0.16%	9,952	1,407,714	0.17%	9,965	1,362,989	0.16%
	MITE-DTC	3,978	545,304	0.06%	3,841	540,512	0.06%	5,185	757,501	0.09%
	MITE-DTH	33,972	4,507,524	0.53%	31,296	4,754,595	0.56%	19,639	3,013,829	0.36%
	MITE-DTM	16,662	5,375,615	0.63%	16,662	5,375,615	0.63%	88,115	14,971,880	1.79%
	MITE-DTT	14,215	1,544,949	0.18%	14,116	1,636,724	0.19%	12,305	1,444,967	0.17%
**DNA TE-RC**										
	Helitron	185,099	56,058,624	6.59%	172,937	55,594,741	6.54%	172,377	49,965,332	5.99%
	Total Transposons	808,869	472,553,091	55.56%	785,512	474,008,142	55.74%	904,510	435,749,018	52.20%
**Tandem Repeats**										
	lm_CentPR	119,412	15,694,985	1.85%	119,412	15,694,985	1.85%	320	17,528	0.00%
	lm_TR	11,895	890,423	0.10%	11,895	890,423	0.10%			
**RNA**										
	rRNA	8,143	5,780,808	0.68%	8,143	5,780,808	0.68%	995	215,445	0.03%
	snRNA	20	2,484	0.00%	20	2,484	0.00%	30	3,233	0.00%
	tRNA	1,777	1,503,980	0.18%	1,777	1,503,980	0.18%	989	218,186	0.03%
	Total Repeats	950,116	496,425,771	58.37%	926,759	497,880,822	58.55%	906,847	436,203,559	52.26%

Supplementary Table 6. Copies and age distribution of full-length LTRs in the little millet genome.

Little millet_JK-8	1 mya	2 mya	3 mya	4 mya	5 mya	6 mya	> 6 mya	Count
Copia_Ale	300	101	21	3	1	0	0	426
Copia_Alesia	20	9	2	0	0	0	0	31
Copia_Angela	18	142	21	4	0	0	0	185
Copia_Bianca	20	26	11	4	0	0	0	61
Copia_Ikeros	14	49	38	10	1	0	0	112
Copia_Ivana	165	37	8	3	0	0	0	213
Copia_SIRE	269	97	11	3	0	0	0	380
Copia_TAR	163	27	10	1	0	0	0	201
Copia_Tork	9	5	8	2	0	0	0	24
Gypsy_Athila	48	109	58	3	0	0	0	218
Gypsy_CRM	476	135	65	6	2	0	0	684
Gypsy_Ogre	0	11	13	1	2	0	0	27
Gypsy_Reina	58	70	18	3	0	0	0	149
Gypsy_Retand	65	262	132	16	0	0	0	475
Gypsy_Tekay	1932	233	38	6	0	0	0	2209
Unclassified	145	206	100	14	2	1	0	468

Broomcorn millet_AJ8	1 mya	2 mya	3 mya	4 mya	5 mya	6 mya	> 6 mya	Count
Copia_Ale	134	97	28	2	1	0	0	262
Copia_Alesia	0	3	12	2	0	0	0	17
Copia_Angela	0	3	2	6	1	0	0	12
Copia_Bianca	2	20	19	4	1	0	0	46
Copia_Ikeros	159	118	42	12	4	0	0	335
Copia_Ivana	90	38	25	2	1	0	0	156
Copia_SIRE	6	16	10	3	1	0	0	36
Copia_TAR	1	2	1	5	0	0	0	9
Copia_Tork	0	5	3	0	1	0	0	9
Gypsy_Athila	0	15	38	9	1	0	0	63
Gypsy_CRM	401	55	41	21	3	0	0	521
Gypsy_Ogre	1485	548	39	18	0	0	0	2090
Gypsy_Reina	91	39	21	1	0	0	0	152
Gypsy_Retand	38	93	120	56	8	0	0	315
Gypsy_Tekay	1503	72	17	4	0	1	0	1597
Unclassified	38	75	87	36	11	3	0	250

Supplementary Table 7. LTR assembly index (LAI) of the little millet genome.

Genome	Size	Intact	Total	raw_LAI	LAI
**Little millet_JK-8**	850,492,432	0.0699	0.4043	17.29	15.33
**Broomcorn millet_AJ8**	834,678,208	0.07	0.45	16.31	14.23

Supplementary Table 8. Structural variants size class distribution across the genome by their type.

Size class	DEL	DUP	INS	INV	Total
**<50 bp**	3,517	-	578	157	4,252
**50-100 bp**	2,280	-	152	196	2,628
**101-1000 bp**	2,849	465	-	253	3,567
**1001-10000 bp**	1,726	338	-	63	2,127
**>10000 bp**	1,050	420	-	229	1,699
**Total**	11,422	1,223	730	898	14,273

Supplementary Table 9. Chromosome-wise structural variants counts by type and total.

Chromosome	Chr_length	DEL	DUP	INS	INV	Total
**Psum.JK8.chr1A**	43,243,216	680	77	54	52	863
**Psum.JK8.chr1B**	43,137,477	551	62	46	46	705
**Psum.JK8.chr2A**	53,162,992	814	67	55	53	989
**Psum.JK8.chr2B**	53,115,015	499	92	28	57	676
**Psum.JK8.chr3A**	61,397,489	951	94	55	58	1,158
**Psum.JK8.chr3B**	50,789,506	530	74	33	49	686
**Psum.JK8.chr4A**	36,975,915	537	49	36	48	670
**Psum.JK8.chr4B**	35,829,819	374	43	29	43	489
**Psum.JK8.chr5A**	47,000,000	588	59	46	55	748
**Psum.JK8.chr5B**	50,323,433	598	51	26	61	736
**Psum.JK8.chr6A**	41,209,881	547	62	21	38	668
**Psum.JK8.chr6B**	37,846,408	488	59	28	38	613
**Psum.JK8.chr7A**	39,612,390	501	58	33	43	635
**Psum.JK8.chr7B**	37,794,386	517	60	34	29	640
**Psum.JK8.chr8A**	41,957,870	1,026	74	73	52	1,225
**Psum.JK8.chr8B**	40,861,194	876	92	63	44	1,075
**Psum.JK8.chr9A**	58,551,545	599	65	27	67	758
**Psum.JK8.chr9B**	57,786,484	714	82	41	62	899
**Psum.JK8.chr0**		32	3	2	3	40
**Total**		11,422	1,223	730	898	14,273

Supplementary Table 10. Structural variants distribution across the gene proximity regions.

SV Type	Within gene	Flank 1K	Flank 2K	Flank 3K	Flank >3K	Total
**Deletion**	1,474	1,937	1,377	902	5,732	11,422
**Insertion**	35	192	135	79	289	730
**Duplication**	529	141	83	46	424	1,223
**Inversion**	356	126	77	66	273	898
**Total**	2,394	2,396	1,672	1,093	6,718	14,273
