# Supplementary material for: Little millet genome reveals evolutionary insights into tetraploid structure and genetic basis of micronutrient density
Source: Nat Commun. 2025 Nov 29;17:74. doi: 10.1038/s41467-025-66716-6 (PMC12769566; doi:10.1038/s41467-025-66716-6)
Supplement: Supplementary file 3 — Description of Additional Supplementary Files [file 41467_2025_66716_MOESM3_ESM.pdf]

Description of Additional Supplementary Files

Supplementary Data 1. Distribution of transcription factor genes in the little millet genome

Supplementary Data 2. List of identifiers encoding transcription factor genes in little millet genome

Supplementary Data 3. Catalogue of Pfam domains identified in little millet genes

Supplementary Data 4. GO annotations and KEGG pathways in little millet genes

Supplementary Data 5. A syntelog matrix representing duplicated homeologues of little millet and broomcorn millet

Supplementary Data 6. Sub-genome dominance patterns of each gene pair across the ten tissues during plant development. Only fully retained gene pairs are selected. The last column shows whether the sub-genome dominance patterns are stable or dynamic across all tissues

Supplementary Data 7. Complete mixture model estimates of Ks distributions

Supplementary Data 8. List of little millet accessions of the resequencing panel

Supplementary Data 9. Summary statistics of read alignments for the diversity panel

Supplementary Data 10. Comparison of geographical origin of the little millet accessions (state-wise) with the population groups identified by different models of populations structure analysis

Supplementary Data 11. Summary of large structural variants identified through LSV-Viz analysis

Supplementary Data 12. BLUP values of agronomic traits and seed mineral concentrations measured in little millet diversity panel

Supplementary Data 13. Marker-trait associations identified in little millet diversity panel using population structure determined by fastSTRUCTURE

Supplementary Data 14. Marker-trait associations identified in little millet diversity panel using population structure determined by ADMIXTURE

Supplementary Data 15. Marker-trait associations identified in little millet diversity panel using population structure determined by DAPC

Supplementary Data 16. Marker-trait associations identified in little millet diversity panel using population structure determined by STRUCTURE

Supplementary Data 17. Significant marker-trait associations identified when fastSTRUCTURE, ADMIXTURE, DAPC and STRUCTURE were used as a covariate in GWAS.

Supplementary Data 18. Marker-trait associations identified in little millet diversity panel using SV markers

Supplementary Data 19. Evaluation of possible phenotypic correlations of LSVs using interquartile range (IQR) outlier analysis

Supplementary Data 20. Distribution of Ionome Genes in Little Millet

Supplementary Data 21. Biosample IDs for raw sequencing data of little millet diversity panel.
