# Supplementary material for: Little millet genome reveals evolutionary insights into tetraploid structure and genetic basis of micronutrient density
Source: Nat Commun. 2025 Nov 29;17:74. doi: 10.1038/s41467-025-66716-6 (PMC12769566; doi:10.1038/s41467-025-66716-6)
Supplement: Supplementary file 25 — Reporting Summary [file 41467_2025_66716_MOESM25_ESM.pdf]

nature portfolio

Corresponding author(s): Sateesh Kagale

Last updated by author(s): Nov 4, 2025

Reporting Summary

Nature Portfolio wishes to improve the reproducibility of the work that we publish. This form provides structure for consistency and transparency in reporting. For further information on Nature Portfolio policies, see our Editorial Policies and the Editorial Policy Checklist.

Statistics

For all statistical analyses, confirm that the following items are present in the figure legend, table legend, main text, or Methods section.

n/a	Confirmed	
☐	☒	The exact sample size (n) for each experimental group/condition, given as a discrete number and unit of measurement
☒	☐	A statement on whether measurements were taken from distinct samples or whether the same sample was measured repeatedly
☐	☒	The statistical test(s) used AND whether they are one- or two-sided<br>*Only common tests should be described solely by name; describe more complex techniques in the Methods section.*
☒	☐	A description of all covariates tested
☒	☐	A description of any assumptions or corrections, such as tests of normality and adjustment for multiple comparisons
☐	☒	A full description of the statistical parameters including central tendency (e.g. means) or other basic estimates (e.g. regression coefficient) AND variation (e.g. standard deviation) or associated estimates of uncertainty (e.g. confidence intervals)
☐	☒	For null hypothesis testing, the test statistic (e.g. F, t, r) with confidence intervals, effect sizes, degrees of freedom and P value noted<br>*Give P values as exact values whenever suitable.*
☒	☐	For Bayesian analysis, information on the choice of priors and Markov chain Monte Carlo settings
☒	☐	For hierarchical and complex designs, identification of the appropriate level for tests and full reporting of outcomes
☐	☒	Estimates of effect sizes (e.g. Cohen's d, Pearson's r), indicating how they were calculated

Our web collection on statistics for biologists contains articles on many of the points above.

Software and code

Policy information about availability of computer code

Data collection	No software was used to collect data for this study.
Data analysis	A multitude of software and databases were used in this study, all of which have been listed, cited, or provided. These include:hifiasm (v0.19.9), HiC-Pro (v3.1.0), 3D-DNA (v180922), EndHiC pipeline (v1.0), Juicebox (v1.11.08), EDTA (v2.2.0), RepeatMasker (4.1.6), Tandem Repeats Finder (TRF v4.09), LTR assembly index (LAI), Quartet (v1.2.2), Braker3, PASA (v.2.5.3), PlantTFDB v5.0, InterProScan version 5.72-103.0, DAGChainer, PanKmer, ClustalW v.2.1, trimAL v.1.2, Phyutility, RAxML v.8.2.12, iTOL v.4, PAL2NAL, DATED, PAML V4.0, Mclust, MCScanX, SynVisio, cutadapt, FastQc, bwa, samtools , bsftools, vcftools, fastSTRUCTURE, adegenet (version 2.1.10), dendextend" R package (version 1.19.0), poppr (version 2.9.6), popLDdecay (version 3.43), Delly (v1.1.6), SURVIVOR, Python (version 3.11.0), GAPIT, LDBlockShow (Version 1.40), SynVisio (Version 1.0), Dated (Version 1.0).

For manuscripts utilizing custom algorithms or software that are central to the research but not yet described in published literature, software must be made available to editors and reviewers. We strongly encourage code deposition in a community repository (e.g. GitHub). See the Nature Portfolio guidelines for submitting code & software for further information.

Data

Policy information about availability of data

All manuscripts must include a data availability statement. This statement should provide the following information, where applicable:

- Accession codes, unique identifiers, or web links for publicly available datasets
- A description of any restrictions on data availability
- For clinical datasets or third party data, please ensure that the statement adheres to our policy

The genome assembly and annotation files are available at Zenodo (https://zenodo.org/records/16776976). Raw sequencing data have been deposited in National Center for Biotechnology Information (NCBI) under the BioProject accession PRJNA1204366 (http://www.ncbi.nlm.nih.gov/bioproject/1204366).

Research involving human participants, their data, or biological material

Policy information about studies with human participants or human data. See also policy information about sex, gender (identity/presentation), and sexual orientation and race, ethnicity and racism.

Reporting on sex and gender	This section does not apply to this study
Reporting on race, ethnicity, or other socially relevant groupings	This section does not apply to this study
Population characteristics	This section does not apply to this study
Recruitment	This section does not apply to this study
Ethics oversight	This section does not apply to this study

Note that full information on the approval of the study protocol must also be provided in the manuscript.

Field-specific reporting

Please select the one below that is the best fit for your research. If you are not sure, read the appropriate sections before making your selection.

[x] Life sciences [ ] Behavioural & social sciences [ ] Ecological, evolutionary & environmental sciences

For a reference copy of the document with all sections, see nature.com/documents/nr-reporting-summary-flat.pdf

Life sciences study design

All studies must disclose on these points even when the disclosure is negative.

Sample size	No statistical methods were used to establish sample size. The samples that were sequenced were selected to represent broad genetic diversity from different provinces in India.
Data exclusions	All sequencing data generated was used in the genome assembly and analyses. Whenever possible, all data was included in the supporting analyses. Data exclusion applies only to field phenotypic data where extreme outliers in the data were detected using the Z-score test and then removed..
Replication	In all analyses that support the genomic and evolutionary analysis, the number of replicates or iterations are indicated in materials and methods or supplemental tables. IThe genome assembliy was validated using multiple methods (i.e. BUSCO, Hi-C and comparions to broomcorn millet and other millet species). Field phenotypic data was generated in 2 locations with three replicates in each location.
Randomization	The randomization for field phenotyping experiment involved the use of alpha lattice design with three replications. Randomization did not directly apply to the genome sequencing and assembly.
Blinding	Blinding does not apply to this study, as the study focuses on genome sequencing.

Reporting for specific materials, systems and methods

We require information from authors about some types of materials, experimental systems and methods used in many studies. Here, indicate whether each material, system or method listed is relevant to your study. If you are not sure if a list item applies to your research, read the appropriate section before selecting a response.

Materials & experimental systems

n/a	Involved in the study	
[x]	[ ]	Antibodies
[x]	[ ]	Eukaryotic cell lines
[x]	[ ]	Palaeontology and archaeology
[x]	[ ]	Animals and other organisms
[x]	[ ]	Clinical data
[x]	[ ]	Dual use research of concern
[ ]	[x]	Plants

Methods

n/a	Involved in the study	
[x]	[ ]	ChIP-seq
[x]	[ ]	Flow cytometry
[x]	[ ]	MRI-based neuroimaging

Dual use research of concern

Policy information about dual use research of concern

Hazards

Could the accidental, deliberate or reckless misuse of agents or technologies generated in the work, or the application of information presented in the manuscript, pose a threat to:

No	Yes	
[x]	[ ]	Public health
[x]	[ ]	National security
[x]	[ ]	Crops and/or livestock
[x]	[ ]	Ecosystems
[x]	[ ]	Any other significant area

Experiments of concern

Does the work involve any of these experiments of concern:

No	Yes	
[x]	[ ]	Demonstrate how to render a vaccine ineffective
[x]	[ ]	Confer resistance to therapeutically useful antibiotics or antiviral agents
[x]	[ ]	Enhance the virulence of a pathogen or render a nonpathogen virulent
[x]	[ ]	Increase transmissibility of a pathogen
[x]	[ ]	Alter the host range of a pathogen
[x]	[ ]	Enable evasion of diagnostic/detection modalities
[x]	[ ]	Enable the weaponization of a biological agent or toxin
[x]	[ ]	Any other potentially harmful combination of experiments and agents

Plants

Seed stocks

Seeds of JK-8 were sourced from the All India Coordinated Research Project (AICRP) on small millets at the University of Agricultural Sciences, Bengaluru, India. Seed material for the diversity panel were sourced from various institutions namely, ICAR-Indian Institute of Millets Research, Hyderabad; College of Agriculture, GKVK, University of Agricultural Sciences, Bangalore; University of Agricultural Sciences, Dharwad; Agriculture College, Dang, Gujarat; and S.G. College of Agriculture and Research Station, Jagdalpur, Chhattisgarh

Novel plant genotypes

This study did not involve novel plant genotypes.

Authentication

No authentication procedures were used in this study.
